# Patterning the Cone Mosaic Array in Zebrafish Retina Requires Specification of Ultraviolet-Sensitive Cones

**DOI:** 10.1371/journal.pone.0085325

**Published:** 2014-01-21

**Authors:** Pamela A. Raymond, Steven M. Colvin, Zahera Jabeen, Mikiko Nagashima, Linda K. Barthel, Jeremy Hadidjojo, Lilia Popova, Vivek R. Pejaver, David K. Lubensky

**Affiliations:** 1 Department of Molecular, Cellular, and Developmental Biology, College of Literature, Science, and the Arts, University of Michigan, Ann Arbor, Michigan, United States of America; 2 Department of Physics, College of Literature, Science, and the Arts, University of Michigan, Ann Arbor, Michigan, United States of America; Purdue University, United States of America

## Abstract

Cone photoreceptors in teleost fish are organized in precise, crystalline arrays in the epithelial plane of the retina. In zebrafish, four distinct morphological/spectral cone types occupy specific, invariant positions within a regular lattice. The cone lattice is aligned orthogonal and parallel to circumference of the retinal hemisphere: it emerges as cones generated in a germinal zone at the retinal periphery are incorporated as single-cell columns into the cone lattice. Genetic disruption of the transcription factor Tbx2b eliminates most of the cone subtype maximally sensitive to ultraviolet (UV) wavelengths and also perturbs the long-range organization of the cone lattice. In the *tbx2b* mutant, the other three cone types (red, green, and blue cones) are specified in the correct proportion, differentiate normally, and acquire normal, planar polarized adhesive interactions mediated by Crumbs 2a and Crumbs 2b. Quantitative image analysis of cell adjacency revealed that the cones in the *tbx2b* mutant primarily have two nearest neighbors and align in single-cell-wide column fragments that are separated by rod photoreceptors. Some UV cones differentiate at the dorsal retinal margin in the *tbx2b* mutant, although they are severely dysmorphic and are eventually eliminated. Incorporating loss of UV cones during formation of cone columns at the margin into our previously published mathematical model of zebrafish cone mosaic formation (which uses bidirectional interactions between planar cell polarity proteins and anisotropic mechanical stresses in the plane of the retinal epithelium to generate regular columns of cones parallel to the margin) reproduces many features of the pattern disruptions seen in the *tbx2b* mutant.

## Introduction

The vertebrate retina is a multilaminar, neural epithelium, with neurons and their processes distributed precisely according to neuronal cell type in the basal layers, and with photoreceptors at the apical surface [1]. Rod and cone photoreceptors are elongated, sensory, neuroepithelial cells; each has a basal axon, a single synaptic terminal, and an elaborate, apical process – a modified ciliated appendage – specialized for visual transduction. Rods and cones express specific visual pigments and sensory transduction components and exhibit specialized neural properties that define their response to light wavelength and intensity. In particular, the different types of cone photoreceptors are distinguished by the specific cone opsin gene they express, which determines their wavelength sensitivity. At the apical epithelial surface (the outer limiting membrane of the retina), rods and cones form adherens junctions with Müller glial cells, whose lamellar processes completely surround each photoreceptor [2], [3]. Rod and cone photoreceptors, with intervening Müller processes, are packed in a quasi-two-dimensional array; the precise nature of the photoreceptor packing varies among vertebrate species and across topographic regions of the same retina.

In teleost fish, the geometric precision of the photoreceptor array is especially well-defined, with strong homotypic and heterotypic correlations leading to a periodic pattern of cone photoreceptor types [4], [5]. For example, zebrafish have four spectrally and morphologically distinct types of cone photoreceptors – designated red, green, blue, and ultraviolet (UV) cones – packed in a crystalline lattice that is built from a 12-cone repeating motif with internal, reiterative, mirror-image symmetry [6], [7], [8], [9], [10]. In addition to expressing specific opsin genes, zebrafish cone types exhibit morphological distinctions in the length of the apical process (red and green cones are longer, blue cones intermediate, and UV cones shorter). Pairs of red and green cones are moreover tightly apposed along the length of their inner segments to form ‘double cones’ [11], [12].

Teleost fish also have the distinctive feature of persistent neurogenesis in the adult retina, which is associated with indeterminate growth [13], [14], [15]. Cone photoreceptors are produced by proliferating retinal progenitors in a circumferential germinal zone at the boundary between neural retina and ciliary epithelium, where successive annuli of new retinal neurons are generated. Cones produced in the germinal zone differentiate as cohorts that are organized as columns a single cell wide and parallel to the retinal margin [10], [16]. Rod photoreceptors are generated secondarily and continuously over a prolonged period within the differentiated adult retina, but not in the germinal zone [15], [17]. Instead, rods originate from specialized progenitors derived from Müller glia located within the inner, differentiated retina, and they are preferentially inserted into the apical epithelium *between* cone columns in the rectangular lattice [10], [18].

The sensory appendages of photoreceptors are elaborated from the apical plasma membrane and contain a connecting cilium, inner segment, and outer segment. Importantly, at the level of inner segments, the membranes of adjacent red, green, and blue cones within a column are directly apposed *without* an intervening Müller glial process [10], [19]. Red, green, and blue cones in adult zebrafish show planar polarized distributions of proteins in the Crumbs (Crb) complex at in their inner segments, with proteins localized at the interface with adjacent cones *within* the column but *not between* columns [10], [19], [20]. The Crumbs complex in the zebrafish retina includes the transmembrane proteins Crb2a and Crb2b and their intracellular scaffolding partners, such as the MMP/Stardust-family member Nok and a related protein, Ponli. Recent work from the Wei laboratory has provided direct and highly convincing evidence that Crb2a and Crb2b mediate homophilic and heterophilic cell-cell adhesion between cone photoreceptors [19], [21], [22]. Although neither the UV cones nor the rod photoreceptors express Crb2b or Ponli, all photoreceptors, as well as Müller glia, express both Crb2a and Nok, which overlap with ZO-1 and other markers of the zonula adherens junctions at the OLM, and are important in the maintenance of epithelial integrity [23]. Crb2a also co-localizes with Crb2b in red, green, and blue cone inner segments, where it has a planar polarized distribution [19]. The cell-cell adhesions mediated by polarized Crumbs complexes create pentameric, single-cell wide units consisting of a blue cone flanked by two pairs of red/green double cones; successive pentamers are separated by a single UV cone to form the columns of the cone lattice. Wei and colleagues have proposed that adhesion mediated by Crumbs complexes could provide a cellular mechanism to organize the cone mosaic pattern [19], [20].

UV cones are distinct from other cone types in several ways: Their inner segments do not extend apically to the level of the other cone inner segments, they do not express Crb2b or Ponli, and the distribution of Crb2a in the apical membrane is not planar polarized. Additional evidence to suggest a lack of planar cell polarity (PCP) is that the position of the basal body and connecting cilium in the plane of the epithelium is random in UV cones, whereas in red, green, and blue cones the basal body is localized asymmetrically on the edge of the cone facing the adjacent column in the direction toward the optic disc, *i.e.* toward central retina [24]. Despite these unique features, UV cones are always positioned correctly within the cone mosaic lattice, but it is not known whether they are important or dispensable in its formation.

In this study we examined cone patterning in a zebrafish genetic model in which UV cone photoreceptors are largely eliminated from the retina by a loss-of-function mutation in *tbx2b*, a member of the T-box subfamily of homeobox transcription factors. Previous studies in zebrafish have shown that Tbx2b is required for proper specification of UV cones; in *tbx2* mutants the number of UV cones is sharply reduced and rod photoreceptors are increased in the embryonic retina [25]. When we analyzed the cone mosaic pattern in adult *tbx2b* mutant zebrafish, we found that the long-range, crystalline organization of linear columns and rows of cone photoreceptors is disrupted in the absence of UV cones, although short-range, planar polarized adhesive interactions between the remaining cones are preserved. We used computer simulations to show that a pattern with these features can be obtained from a model in which UV cones initially begin to differentiate at the retinal margin, but are then rapidly eliminated before the ordered cone lattice has been established. This is consistent with our observation that a few misformed UV cones can be identified near the margin, but they are eliminated as the cone mosaic matures.

## Materials and Methods

### Animals

Zebrafish (*Danio rerio*) were maintained at 28°C on a 14/10 hour light/dark cycle with standard husbandry protocols [26]. All studies reported here were carried out in accordance with the recommendations in the Guide for the Care and Use of Laboratory Animals of the National Institutes of Health. The University Committee on the Use and Care of Animals at the University of Michigan approved all protocols.

Transgenic zebrafish lines with reporters for specific opsin genes included: *rhodopsin Tg(-3.7rh1:EGFP)ki2* [27], *UV cone opsin Tg(-5.5sws1:EGFP)kj9* [28], *blue cone opsin Tg(-3.2sws2:mCherry)mi2007* [10], and *Tg(trβ2:tdTomato)* [29]. Alternative gene names for these opsins are *sws1 = opn1sw1*, *sws2 = opn1sw2*, and *rh1 = rho*. The transgenic lines used are henceforth referred to as rod reporter, UV cone reporter, blue cone reporter, respectively.

The mutant allele *tbx2b^c114^* [30] was crossed into one or more of these transgenic photoreceptor reporter lines. We discovered that the *-3.2sws2:mCherry* transgene was genetically linked to the *tbx2b* gene on chromosome 11, so embryos from *tbx2b^−/−^*×*Tg(sws2:mCherry)* crosses were screened for genetic recombination to recover the *tbx2b* mutant allele linked with the blue cone opsin reporter. The calculated recombination rate was 0.008. We found that the *txb2b^c114^* homozygous mutant adult is semi-viable and infertile, so the *sws2:mCherry* transgene could not be stably introduced into the mutant background. Mutants with the blue cone reporter therefore had to be recovered by recombination from *tbx2b^+/−^;sws2:mCherry* heterozygous in-crosses.

To destroy photoreceptors, freely-swimming fish were exposed to ultra-intense light (>160,000 lux) for 30 minutes as previously described [31].

### Tissue preparation

Embryonic fish at 3 to 4 days post-fertilization (dpf) were anesthetized with 0.2 mg/ml Tricaine (Sigma-Aldrich A5040), and euthanized by decapitation. The heads were fixed in 4% paraformaldehyde in 0.1M phosphate buffer (pH 7.4) with 5% sucrose overnight at 4 C, rinsed, and processed for immunocytochemistry as described below.

For adult retinal flat-mount preparations, zebrafish were placed in the dark for 30 minutes and then anesthetized in Tricane (Sigma-Aldrich A5040) and euthanized by cervical dislocation. The eyes were enucleated and a radial cut was made along the ventral axis of the eyecup for orientation. The entire anterior segment was removed with microscissors, and the eyecup was placed in phosphate buffered saline (PBS), while the neural retina was gently removed from the retinal pigmented epithelium. Short relaxing cuts were made along the perimeter and isolated retinas were fixed in 4% paraformaldehyde in 0.1M phosphate buffer (pH 7.4) with 5% sucrose overnight at 4 C, rinsed, and processed for immunocytochemistry as described below.

For cryosections, tissue was prepared as described previously [32].

### Immunocytochemistry

Immunocytochemistry on retinal flat-mounts and embryonic heads was performed as previously described [10], or with slight modifications. For antigen retrieval, retinas were placed in sodium citrate buffer (10 mM sodium citrate, 0.05% Tween 20, pH 6.0), and incubated in boiling water for 10 minutes, removed from the heating plate, and allowed to cool in the hot water for 10 minutes. Tissues were washed with PBS/0.5% Triton-X100 and then blocked for 2 hours in 10% normal goat serum, 1% Tween, 1% Triton X-100, 1% DMSO, in phosphate buffered saline with 0.1% sodium azide. Incubation in primary antibodies was overnight at room temperature with the following dilutions: mouse anti-Zonula Occludens-1 (Invitrogen Corporation, Camarillo, CA), 1∶200; rabbit anti-Crb2b (gift of Xiangyun Wei, University of Pittsburgh School of Medicine), 1∶200; zpr1, Zebrafish International Research Center, 1∶400. Zpr1 is a monoclonal antibody that recognizes cone arrestin 3a, which is expressed specifically by both red and green cones [33]. After washing four times in phosphate buffered saline with 1% Tween, 1% Triton X-100, 1% DMSO, tissues were incubated with anti-mouse DyLight 549 and/or anti-rabbit DyLight 647 secondary antibodies (Jackson ImmunoResearch Inc., West Grove, PA) overnight at room temperature. Isolated retinas were rinsed and mounted on a microscope slide, typically with the photoreceptor side down, in Prolong Gold (Invitrogen). Eyes from the 3–4 dpf embryonic heads were removed and mounted pupil down on a microscope slide with Prolong Gold.

Immunocytochemistry on 6 µm retinal cryosections was performed as previously described [10], with the exception that incubation with the primary antibody was overnight at 4°C, and nuclei were stained with Hoechst.

### RT-PCR

Total RNA was isolated from adult retinas using TRIzol reagent (Invitrogen); cDNA was synthesized with the SuperScript III First-Strand Synthesis System for RT-PCR (Invitrogen) and RT-PCR was performed with Taq DNA Polymerase (5 PRIME, Inc., Gaithersburg, MD) and specific primers – *rho*: forward-GGCTTCACCACCACCATGTA, reverse-GTGGTGATCATGCAGTGACG; *opn1sw1*: forward-TCCAGCAAGACCGAAACCTC, reverse-ACAGGAGCAGACAGTGAACG; *opn1sw2*: forward-GGGACACAGTGGCGTATTCA, reverse-AGCTTGAGCTTTGGCTGCTA), *gpia*: forward-GCGTATTTCCAACAGGGGGA, reverse-GTCTCCGGACAACCCAGAAG.

Three biological replicates were tested for wild-type siblings and *tbx2* mutant retinas. Intensity of the bands was measured with ImageJ.

### Image acquisition and processing

Images were collected on a Zeiss Axio Image ZI Epifluorescent Microscope with ApoTome attachment (Carl Zeiss Microimaging Inc., Thornwood, NY), or a Leica SP5 Scanning Confocal Microscope (Leica Microsystems, Bannockburn, IL), or an Olympus FluoView 500 Laser Scanning Confocal Microscope (Olympus America Inc., Center Valley, PA), or a Nikon A1 Spectral Confocal Microscope (Nikon Instruments, Inc., Melville, NY). Post-acquisition processing was done with Adobe Photoshop CS5 Extended (Adobe System Inc., San Jose, CA) or ImageJ (*rsbweb.nih.gov*). The fluorescent signals captured by specific channels were sometimes pseudocolored for better visibility and for ease of identification to reflect the wavelength specificity of the specific photoreceptor types, *i.e.*, green for rods, blue for blue cones, magenta for UV cones.

### Photoreceptor counts

Whole eyes (3 to 4 dpf embryos) or isolated retinas (adults) from wild-type and *tbx2* mutants crossed into the transgenic rod reporter line or both the UV cone and blue cone reporter lines were immunostained with Zonula Occludens-1 (ZO-1) antibodies. Confocal images were collected from the central retina of embryonic eyes at 3 dpf and from the ventral hemisphere of adult retinas. Adobe Photoshop was used to generate a flattened projection of ZO-1 labeling in the zonula adherens junctions between photoreceptors and Müller glia at the level of the outer limiting membrane (OLM); the ZO-1 label outlines the apical membrane profiles of the rod and cones. Profiles of rods, blue or UV cones in the respective transgenic lines were identified by presence of the specific fluorescent reporter inside the cell at the level of the ZO-1 label. The layer masking function in Photoshop was used to isolate the transgene signal within the proper cell profile at the level of the ZO-1 label. Localization of the transgene in the ZO image was confirmed by the scrolling through the original z-stack.

To determine planimetric densities (#/10^3^ µm^2^) of rods and cones in embryonic and adult retinas from wild-type and mutant fish, the rod reporter line was used and rod and cone photoreceptors were counted separately in an area of approximately 870 µm^2^ (embryonic retina) or 3,200 µm^2^ (adult retina) in the plane of the OLM. To avoid over-sampling, at the boundaries of the counting window, only ZO-1 profiles that touched two of the four sides were included. ImageJ was used for counting; data were analyzed for significance with Student's t-test, and plotted with SigmaPlot software (Systat Software Inc., http://systat.com/).

To determine the ratio of blue cones to double cones (red/green pairs) in adult retinas from wild-type and mutants, samples from fish carrying both the UV cone and blue cone reporters were analyzed from the ventral hemisphere in retinal flat-mounts immunolabeled with ZO-1. The dorsal hemisphere was not sampled because the red/green cone profiles in this region are smaller than in ventral retina and cannot be reliably distinguished from the profiles of rods. Each sample counted contained between 40 and 80 blue cones, and the ratio of red/green cones to blue cones was calculated; data were analyzed for significance with Student's t-test.

### Image segmentation and identifying adjacent cones

Retinal flat-mounts immunostained with anti-ZO-1 from wild-type and mutant rod reporter adult fish were imaged in the ventral retinal region. The fluorescent reporter driven by rhodopsin allowed us to unambiguously distinguish rods from cones. We first used the ZO-1 signal to identify each cell: We extracted the data in the ZO-1 channel from the raw image z-stack and projected it to a single image using ImageJ; we then defined individual cell profiles by performing thresholding-based image segmentation in MATLAB. To determine which of these cells were rods, we next identified for each cell the single z-slice with the highest average ZO-1 intensity around that cell and calculated the average EGFP (rod reporter) intensity in that slice and within the given cell. We then used a k-means algorithm to divide these average intensities into two clusters. The ZO-1-delimited, rounded cell profiles with little or no EGFP signal were designated as cones; irregular profiles that we showed previously to be characteristic of Müller glia [10] were excluded.

To identify neighboring cones, we relied on the observation that two adjacent cones not only are closely separated but tend to share a length of common interface. To quantify this feature, we defined the region between two cells by applying a morphological closing algorithm with a circular structuring element of radius 10 pixels on every pair of segmented cells [34]. We characterized the size and shape of this region by calculating its moment of inertia tensor. We defined the thickness of the region (roughly the distance between the two cells) as the smaller of the two principal moments of inertia and took the ratio of the two principal moments as a measure of how elongated the region is. These two numbers were used in defining our adjacency criteria. To avoid over dependency of the result on the parameters chosen, we tried to establish upper and lower bounds using two sets of parameters. For the low threshold parameters, which were designed to identify all possibly adjacent pairs while tolerating inclusion of false positives, we required a maximum thickness of 10 pixels and a minimum ratio of 2 for two cells to be considered adjacent. On the other end of the spectrum, for the high threshold parameters designed to exclude almost all non-adjacent pairs while potentially omitting some true pairs, we used 10 pixels and 2.5 for the maximum thickness and minimum ratio, respectively. The image magnification was 0.1 µm/pixel (Zeiss ApoTome, 63× oil immersion objective).

### Mathematical model

The details of the mathematical model employed in this paper can be found in our previous publication [10], in which we modeled the cone mosaic observed in the wild-type zebrafish retina. To study the disrupted cone patterning seen in the retinas of *tbx2b* mutants, we made the following modifications:

#### Initial conditions

A few columns of cone photoreceptors were initially established by progressive growth of the cone pattern with the previous model [10]. A new column of cone cells with random distribution of planar cell polarity proteins was then introduced at the interface between the established cone columns (with polarized proteins) and the precursor cells (without proteins). In this column, uniformly spaced cells with approximately five cells between them were marked as UV cones. In the subsequent introduction of new columns adjacent to the previously established columns, the cells in the new column with centroid closest to the midpoint between the centroids of the two UV cells in the previous column were marked as UV cone cells. These UV cells were initialized with random distribution of proteins similar to the other cone cells.

#### Elimination of UV cones

The tension λ_0_ along the edges of the presumptive UV cone cells was increased by threefold in comparison to the neighboring cone cells. The preferred area A_0_ of the UV cone cells was reduced gradually by changing 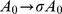, where the shrink factor 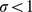, at regular time intervals 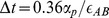. The tuning of the shrink factor 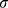 enabled the tuning of the relaxation time of the shrinking UV cell. This was accompanied by a simultaneous reduction of PCP proteins on the edges such that the total number of polarity proteins in a given, shrinking cell changed by 
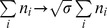
, where 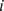 runs over the edges of the cell. This ensured that the concentration of polarity proteins remained finite on the shrinking edges. The packing as well as the PCP protein concentrations were evolved in the intervals between the reductions in preferred area and PCP proteins. As the area of the UV cells decreased on evolving the packing, the cells lost their edges by successive T1 transitions [35] until they were removed on reaching a minimum cutoff area.

#### Topological transitions

We modified the procedure we described previously [10] for T1 topological transitions to allow for the orientation of the new edge to depend on the tensions in the four existing edges and to ensure that the PCP protein concentrations on the new edge are reasonable even when the four existing edges have very different concentrations.

After the collapse of an edge into a 4-fold vertex, two possible topologies, depending on the two pairs of edges chosen to be adjacent to each other, were tested for favoring a T1 transition (see Fig. S5B in [10] for notation). In each of these topologies, the orientation of the possible new edge was determined by examining the forces exerted by the chosen adjacent edges on the 4-fold vertex. To illustrate this, consider the first possible topology in which edges 1 and 3 meet in one vertex and edges 2 and 4 meet in the other. The forces exerted by these two pairs of edges on the 4-fold vertex are 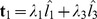 and 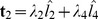 respectively. Here 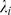 represents the tension on edge *i* (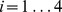), and 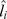 is the unit vector tangent to edge *i* at the vertex. The orientation of the new edge was chosen to be parallel to 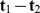. The edges were then then allotted polarity proteins according to
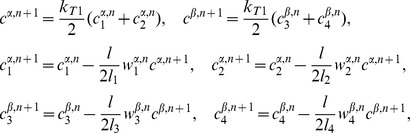
where superscript *n* and *n*+1 label the concentrations immediately before and after the *n* ^th^ T1 transition, superscript 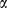 and 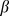 denote the two cells separated by the new edge, and quantities without a numerical subscript *i* (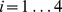) refer to the newly created edge. Here 
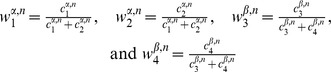
. In the simulations, we chose 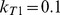.

#### Parameters

The parameters of this model were normalized as described in [10] using a reference tension 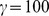 and a reference length *l* satisfying 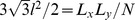, where 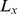 and 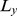 are the lengths of the box in the *x* and *y* directions (after compression perpendicular to the margin) and *N* is the total number of cells. We used the following parameter values: 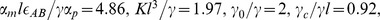 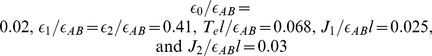. These parameters lie within the range in which the model did not show any qualitative change in patterns in the previous publication [10]. The shrink factor for the preferred area in the UV cone cells 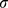 was chosen to be 0.4.

## Results

### UV cones are reduced in number and dysmorphic in *tbx2b* mutants

We began by characterizing the embryonic retinal photoreceptor phenotype in zebrafish *tbx2b* mutants homozygous for the *c144* allele [30]. A previous analysis of the *tbx2b^c144^* allele characterized disruptions in patterning of the parapineal in the brain and identified a premature stop codon near the C-terminal end of the DNA-binding domain (T-box). Microinjection of *tbx2b* antisense morpholinos into *tbx2b^c144^* mutants did not enhance the mutant phenotype in the parapineal, suggesting that this mutation is a functional null [30].

**Figure 1:**
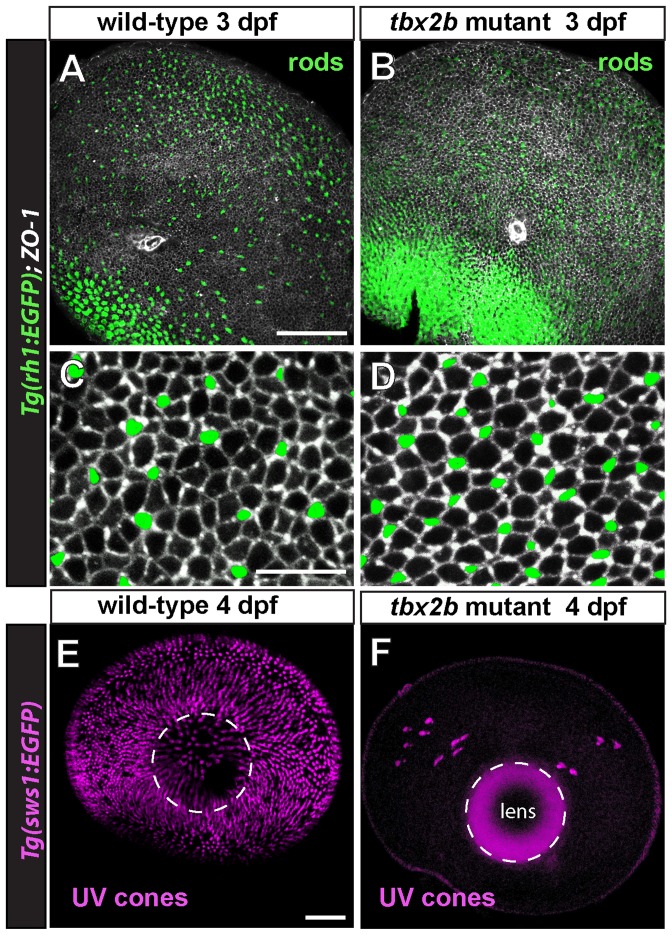
Rod photoreceptors develop rapidly and UV cones are missing in *tbx2b* mutants. A) Isolated, larval *rh1:EGFP* eye at 3 days post-fertilization (dpf) viewed from the scleral aspect. Rod photoreceptors are green and immunostaining for the apical junctional marker, Zonula Occludens-1 (ZO-1) is in white. Dorsal is up; the optic nerve appears as a small white ring ventral to the center. B) Mutant *tbx2b; rh1:EGFP* larval eye at 3 dpf viewed from the scleral side. Note increased number of rods, especially in the ventral retina. C, D) Higher magnifications of central retina in wild-type and mutant eyes, respectively. E) Isolated, larval *sws1:EGFP* eye and F) *tbx2b; sws1:EGFP* eye at 4 dpf, viewed from the scleral aspect. Cones expressing the UV opsin reporter are pseudocolored magenta. The lens, outlined with a dashed white line, shows background fluorescence in F, due to the longer exposure time required to capture immunofluorescence of the few scattered UV cones. Scale bars:  = 50 µm (A,B); 10 µm (C,D); 50 µm (E,F).

To examine rod photoreceptor development and patterning in the retina, we examined fish in which the mutant allele *tbx2* was crossed into the rod reporter line, which expresses EGFP in rod photoreceptors. Enucleated eyes are viewed from the back (scleral side) at 3 dpf and immunostained with anti-Zonula Occludens-1 (ZO-1), an adherens junction protein that marks the boundaries between cells at the apical retinal surface, known as the outer limiting membrane, OLM [10]. The small white ring of ZO-1 staining near the center in Figures 1A, B is the optic disc. In wild-type embryos (Fig. 1A), EGFP-labeled rods in the reporter line are at highest density in the precociously developing ‘ventral-nasal patch’ and are scattered across the remainder of the retina, with a gap in central retina that is quickly filled in over the next few days as more rods continue to be generated across the retina [36], [37], [38]. In homozygous *tbx2b* embryos at the same developmental stage, EGFP-labeled rods are more abundant in the ventral-nasal patch as well as across the remainder of the retina (Fig. 1B). The distribution of rods in these mutant retinas at 3 dpf is similar to the pattern observed a few days later in wild-type zebrafish [37], suggesting that rod differentiation is accelerated in the mutant and/or the rate of production of rods is increased.

**Figure 2:**
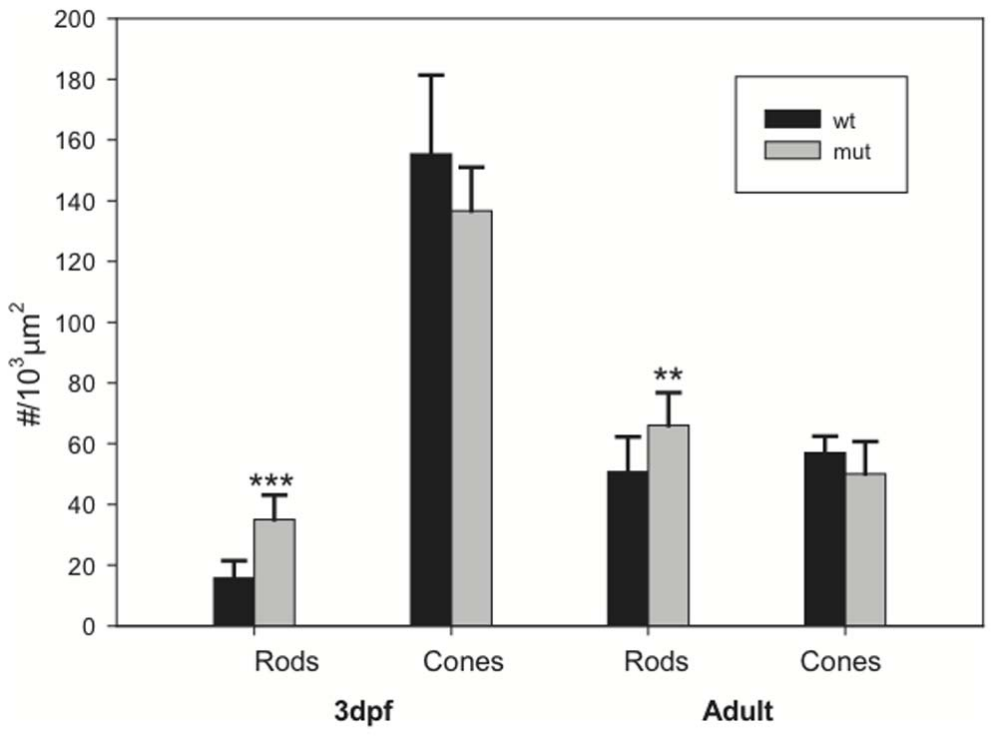
The rod phenotype in *tbx2b* mutant embryos is partially compensated in adults. Rods and cones were counted in wild-type (wt) and *tbx2b* mutant (mut) embryos at 3 days post-fertilization (dpf) and in adult retinas and plotted as planimetric density (#/10^3^ µm^2^). Photoreceptor profiles were identified by ZO-1 immunostaining and the rod opsin transgenic reporter was used to distinguish rods. Means ± standard deviation are plotted for n = 11 samples from 3 retinas (wt embryo), n = 8 samples from 4 retinas (mut embryo), n = 12 samples from 3 retinas (wt and mutant adult). *** p<0.001; ** p<0.01.

Apical cell profiles of individual cones and rods are visualized by the adherens junction marker, ZO-1, shown in higher magnification images from a central region, just dorsal to the ventrally-displaced optic disc in embryonic retinas of wild-type siblings (Fig. 1C) and *tbx2b* mutants (Fig. 1D). Images from four mutant and three wild-type siblings were used to quantitate rod and cone size and planimetric density. Figure 2 shows that *tbx2b* mutant embryos have over twice as many rods (35±8.1 rods/10^3^ µm^2^) as wild-type embryos (16±5.7 rods/10^3^ µm^2^; p<0.001, n = 4 mutant and 3 wild-type). The average planimetric density of cones at the OLM was slightly lower (∼12%) in *tbx2b* mutants (140±14 vs. 160±26 cones/10^3^ µm^2^), but the difference was not significant. Although the average planimetric density of cones is not different in *tbx2^c144^* mutant embryos, a previous phenotypic analysis of a different mutant allele (*tbx2b^p25bbtl^*) reported that UV cones are largely absent [25]. To identify UV cones, we examined fish in which the *tbx2^c144^* allele was crossed into the UV cone reporter (EGFP) line, and we confirmed that the *tbx2b* mutant retinas have very few UV cones at 4 dpf (Fig. 1 E, F).

**Figure 3:**
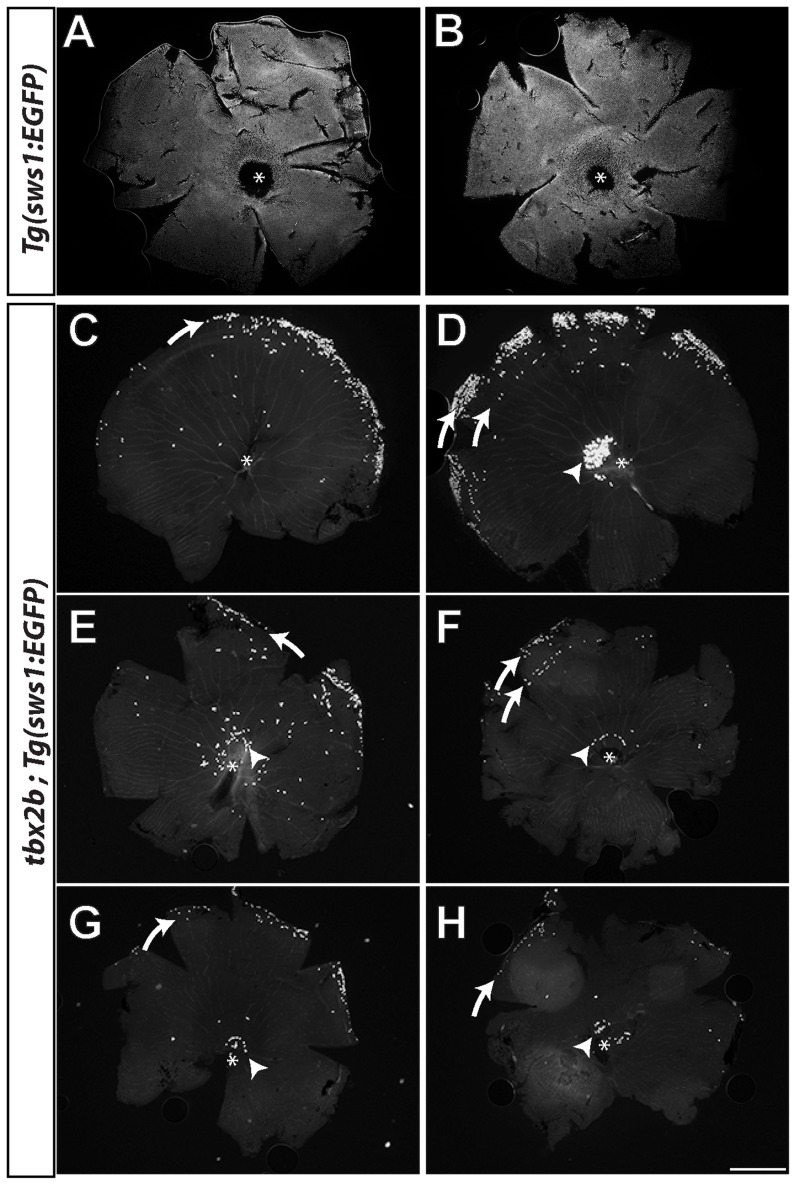
Variable number and distribution of UV cones in adult *tbx2b* mutants. A, B) Flat-mounted retinas from *sws1:EGFP* wild-type fish; the optic disc is indicated by an asterisk; dorsal is up. C–H) Six examples of *tbx2b; sws1:EGFP* mutant retinas. Residual UV cones are typically at or near the dorsal margin (curved white arrows) and immediately dorsal to the optic disc (arrowhead). Scale bar: 500 µm (A–H).

As expected from the embryonic phenotype, UV cones were also rare in adult *tbx2b^c144^* homozygous mutants, but different individuals showed highly variable numbers of residual UV cones (Fig. 3). The two panels at the top (Fig. 3A, B) are retinal flat-mounts (left and right eyes, respectively) from a wild-type, transgenic adult fish with the UV cone reporter. The six panels at the bottom (Fig. 3C–H) are the right and left eyes of three *tbx2b* mutants crossed into the UV cone reporter line. Note that five of the six mutant retinas (Fig. 3D–H) have a small patch of UV cones in the center of the retina, typically arranged in an arc adjacent to the optic nerve (arrowheads). This area around the optic nerve represents the oldest retina, called the ‘larval remnant’ because these cells were present in the embryonic and larval fish [6]. As the eye grows during post-larval life, new neurons, including cones, are added in concentric rings at the retinal periphery in a circumferential germinal zone [6], [10], [16]. As a consequence, age of cone photoreceptors is proportional to distance from the optic disc, with those near the center generated during embryonic or larval life.

All six of the retinas from *tbx2b* mutant adult fish also have scattered UV cones near the retinal margin, but curiously only in the dorsal hemisphere (Fig. 2C–H, curved arrows); in two of the mutant retinas (Fig. 3D, F), UV cones are found in two concentric, partial rings near the margin. The topographic pattern of UV cones in the mutant is puzzling: UV cones present in the adult retina represent both the ‘oldest’ (*i.e.*, present in the embryonic/larval retina) and the ‘youngest’ (*i.e.*, those most recently generated by the circumferential germinal zone, but only in dorsal retina). These observations suggest that in the mutant: 1) some UV cones generated in the embryonic/larval retina survive, and 2) variable numbers of UV cones are generated sporadically in the germinal zone but only on the dorsal side of the retina. Since UV cones are seen at the dorsal margin in adult *tbx2b* fish up to 9-months-old, we assume that UV cones are sporadically generated at the dorsal margin throughout post-larval growth. If this is correct, then the addition of new retina at the margin might be expected to result in a sector (pie-wedge-shape) of UV cones from the optic disc radiating toward the dorsal rim. Instead, however, the distribution of UV cones is discontinuous, including the most recently generated, dorsally located UV cones and the dorsally located UV cones that had differentiated in the embryonic/larval retina, but none in between. The implication of these patterns is that UV cones added successively to the retina during post-larval growth must eventually be eliminated.

We therefore asked whether UV cones die in *tbx2* mutants. However, we found no evidence of apoptosis in the photoreceptor layer or at the retinal margin (data not shown). We examined TUNEL labeling in cryosections from adult *tbx2* mutants (n = 4 retinas from 2 fish) and activated caspase in retinal flat-mounts (n = 6 retinas from 4 fish). As a control for the analysis of apoptosis, we processed retinal sections or whole mounts from wild-type fish exposed to an intense light that destroys photoreceptors, and we found abundant TUNEL and activated caspase labeling at 12 hours post-lesion as previously demonstrated [31]. Although we found no direct evidence for apoptosis of UV cones in *tbx2b* mutants, we cannot be certain that apoptosis does not occur, since TUNEL and anti-caspase labeling is very transient in dying cells. Another possibility that we cannot rule out is that the UV cones might be actively extruded from the epithelium; cell extrusion from epithelia, either with or without cell death, has been characterized in developmental morphogenesis and epithelial homeostasis [39], [40].

**Figure 4:**
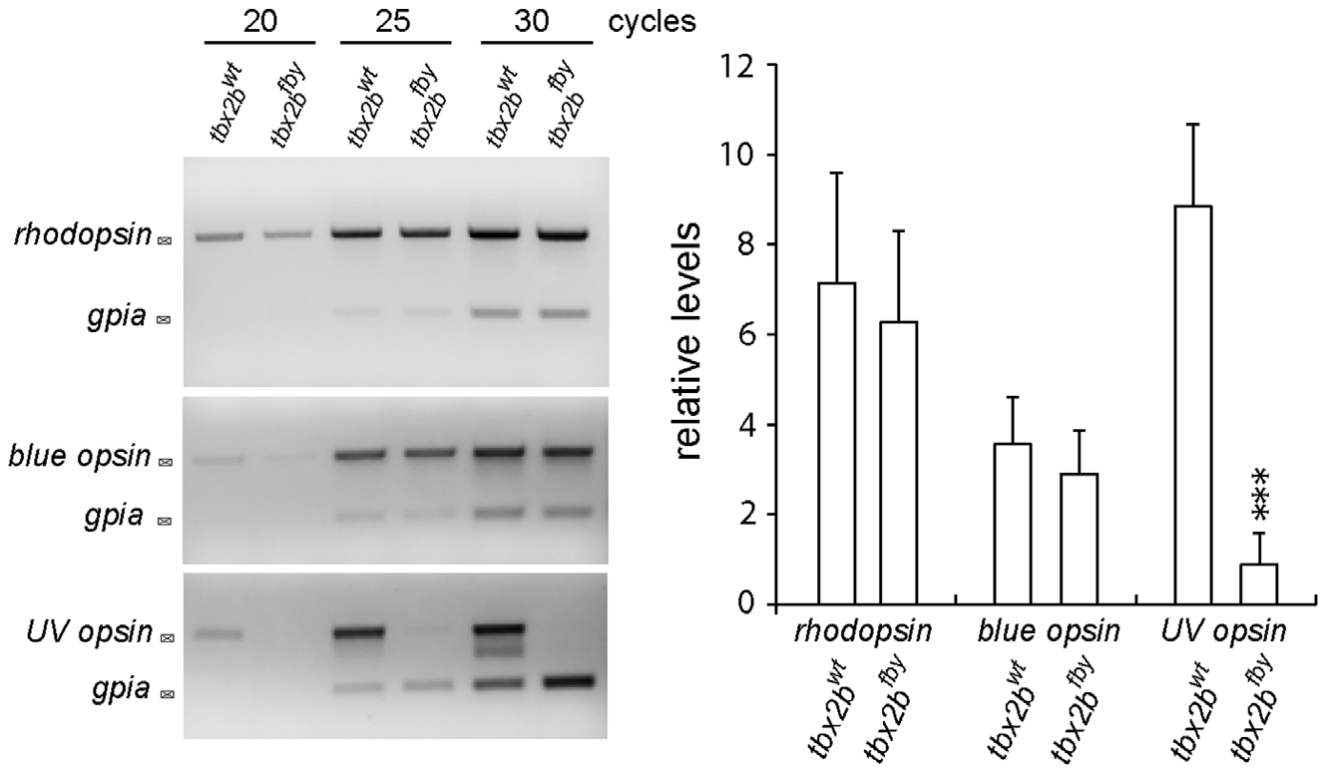
Expression of UV opsin message is reduced in adult *tbx2b* mutants. A) Semi-quantitative RT-PCR of isolated, whole retina from adult wild-type and *tbx2b* mutant fish. Amplification of *rhodopsin (rh1)*, *blue opsin (sws2)*, and *UV opsin (sws1)* mRNA, with *gpia* as a loading control, after 20, 25, and 30 cycles. B) Average levels of expression relative to *gpia*. Three replicates with two retinas (right and left eyes) per sample. *** p<0.001.

To confirm that the apparent absence and/or elimination of UV cones was not due to misregulation of the EGFP reporter driven by the *sws1* UV opsin promoter, while the UV cones survived undetected in the UV cone reporter line, we used semi-quantitative RT-PCR to measure the level of rhodopsin (*rh1*), UV cone opsin (*sws1*), and as a control, blue cone opsin (*sws2*) mRNA (Fig. 4A). We found that in *tbx2b* mutant adult retinas, the relative levels of rod and blue opsin mRNA expression were not significantly different from siblings, but UV cone opsin was very significantly reduced (Fig. 4B, p<0.0001).

The lack of increased levels of rhodopsin mRNA message in the *tbx2b* adult retina is different from what would be expected if rods are more abundant in the mutant. So, we next used the rod reporter line and immunostaining with ZO-1 on retinal flat-mounts from sibling and *tbx2b* mutant fish to assess whether rods continued to be present in increased numbers in the adult mutant retina. We counted rods and cones (not identified by type) in ventral retina (Fig. 2) and found that rods were increased in the mutant by about 30% compared with wild-type (66±10.7 rods/10^3^ µm^2^ vs. 51±11.5 rods/10^3^ µm^2^; p<0.01), whereas cone density was slightly decreased but not significantly (50±10.7 cones/10^3^ µm^2^ vs. 57±5.4 cones/10^3^ µm^2^). Together these results indicate that the predominant phenotype of the *tbx2b* mutant with respect to photoreceptor patterning is a profound loss of UV cones, where UV cone identity is defined by expression of UV opsin. Assuming that rods in the *tbx2b* mutant express an equivalent level of rhodopsin, the adult phenotype includes a modest increase in number of rods.

**Figure 5:**
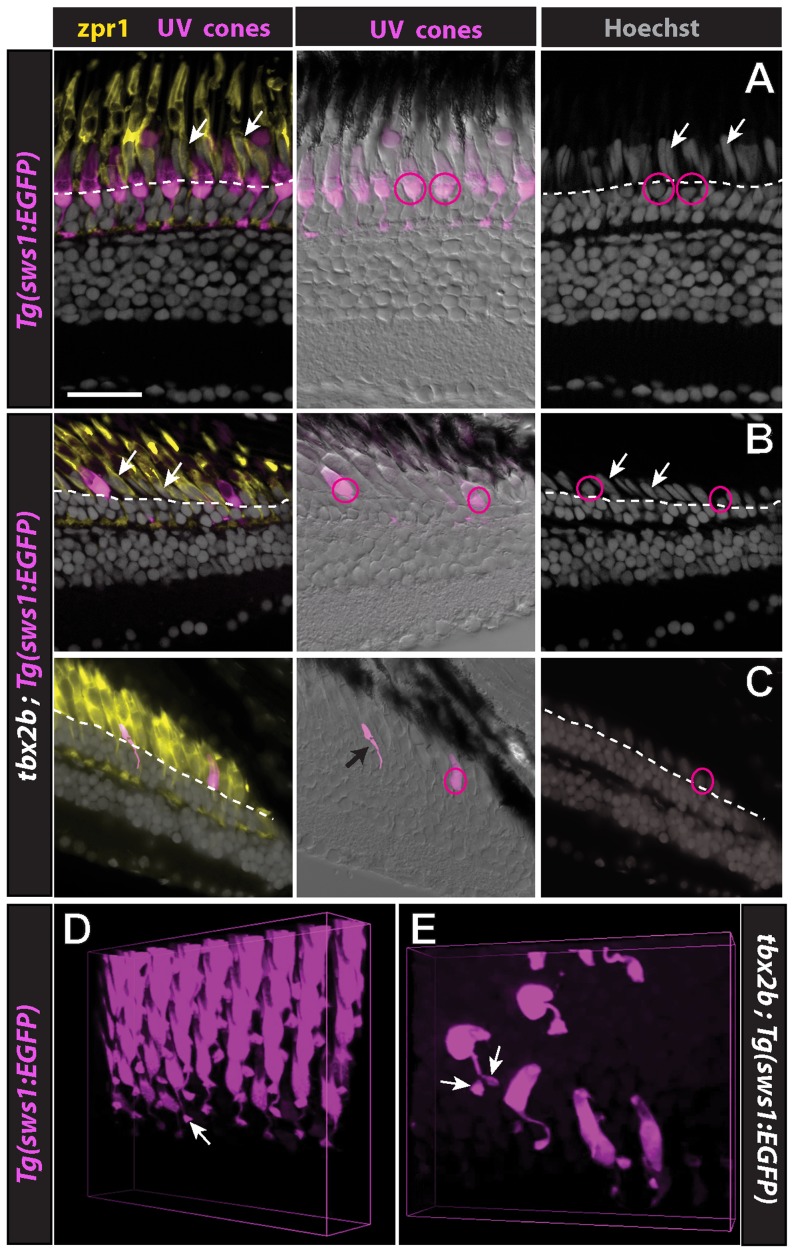
UV cones in *tbx2b* mutants are dysmorphic. A) Retinal cryosection from a *sws1:EGFP* adult zebrafish; UV cones are magenta, red-green double cones are immunostained with the specific marker, zpr1 (yellow), and nuclei are stained with the fluorescent dye, Hoechst (grey). The central panel is an overlay of the epifluorescent channel (UV cones) and transmitted light (differential interference contrast). Nuclei of red-green double cones (arrows), and blue cones (not indicated), are radially elongated and displaced apical to the outer limiting membrane (dashed line), whereas nuclei of UV cones are triangular in shape (circles) and positioned basal to the outer limiting membrane. B, C) In *tbx2b; sws1:EGFP* mutants, the morphology of red-green double cones (zpr1, yellow) and their nuclear position (white arrows) is normal, whereas the nuclei of UV cones (circles) are displaced apically beyond the outer limiting membrane (dashed line). One UV cone (black arrow) is collapsed. D, E) 3D volume renderings of UV cones near the retinal margin in wild-type, *sws1:EGFP* fish. Like all photoreceptors UV cones have a single, bulbous axonal terminal (cone pedicle, arrow). D) Many UV cones in *tbx2b; sws1:EGFP* mutant adults have a bifurcated axon and two pedicles (arrows). Scale bar: 20 µm (A,B,C).

The rare UV cones that survive in dorsal retina of the *tbx2* mutant are dysmorphic. In wild-type zebrafish retinas, the UV cones have a distinctive size, shape, and position – their apical process is much shorter and their nuclei are located entirely basal to the OLM (Fig. 5A, circles) in contrast to the other cone types (red/green double cones and blue single cones), which are more elongated with apically displaced nuclei (Fig. 5A, arrows) [7], [41]. The nuclei of UV cones in adult *tbx2* mutants are displaced apically, sometimes completely beyond the OLM (Fig. 5B, C circles b), and a few UV cones were severely malformed (black arrow in Fig. 5C). We examined 52 radial retinal cryosections through the dorsal-ventral axis of six *tbx2b* mutant fish and found a total of 28 UV cones; the nucleus was apically displaced in 26 of them.

The UV cones in adult *tbx2* mutants also have abnormal axonal (basal) processes. Cone axons in wild-type fish terminate in a single synaptic structure called a cone pedicle (Fig. 5D and Movie S1), but in the *tbx2* mutants some of the UV cone axons have unusual kinks and 11% (27 of 246 UV cones examined in 5 mutant retinas) bifurcate and form two cone pedicles (Fig. 5E and Movies S2 and S3). In contrast, the red and green cones in the adult *tbx2* mutants have normal morphology, as shown by zpr1 staining (compare Fig. 5A with Fig. 5B,C).

### Long-range crystalline order of the cone mosaic pattern is disrupted in *tbx2b* mutants

**Figure 6:**
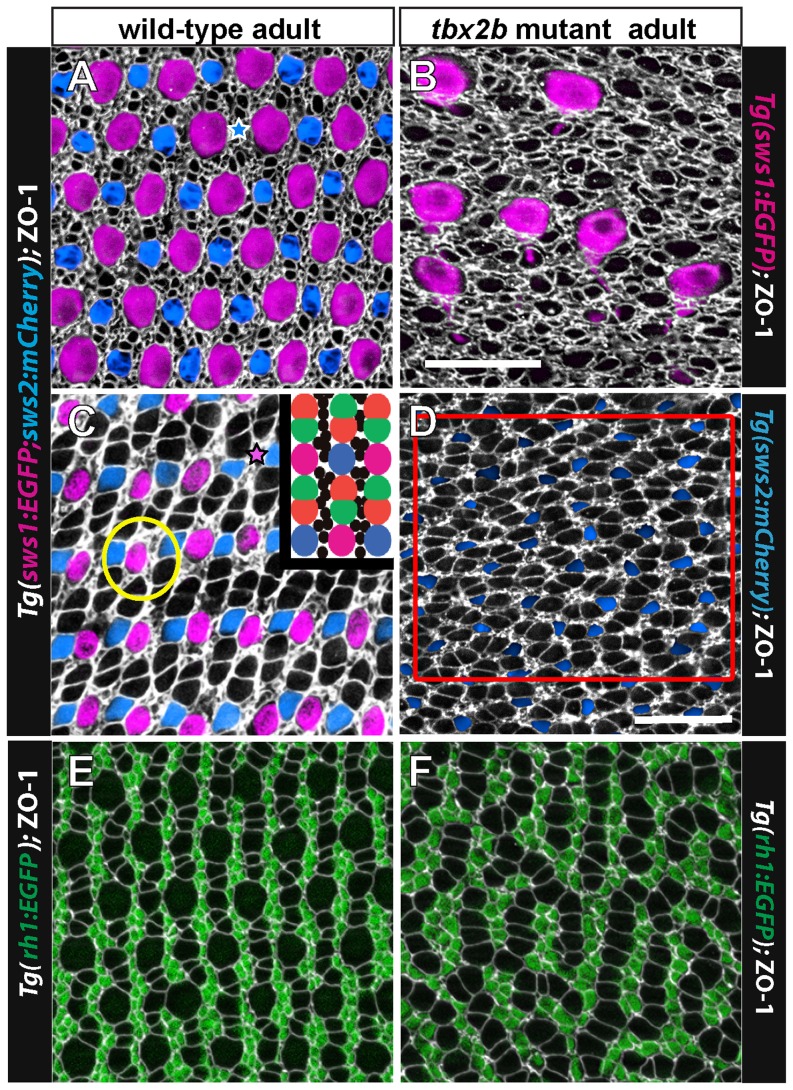
The regular cone mosaic lattice is disrupted in *tbx2b* mutants. A, C) Cone mosaic lattice in the dorsal retina (A) and ventral retina (C) visualized in retinal flat-mounts of adult, wild-type, double transgenic (UV and blue cone reporter) fish. Cell profiles are stained with anti-ZO-1 (white). Rows of UV cones (magenta) and blue cones (blue) alternate with red-green double cones. Smaller, rounded ZO-1 profiles represent rods and irregular profiles are Müller glial processes. The inset shows a cartoon of the cone pattern; rods are represented by black dots. Rarely, cones are missing from the lattice (indicated by blue star in A and magenta star in C). The yellow circle encloses a UV cone surrounded by rods. B) Retinal flat-mount near the dorsal retinal margin of a *tbx2b* mutant with the UV cone reporter. Immunolabeling with ZO-1 (white) reveals absence of a rectilinear order, independent of the presence of the unusually large UV cones (magenta). This fish did not carry the blue opsin reporter transgene. D) Retinal flat-mount from ventral retina in a *tbx2b* mutant double transgenic: blue cones (blue) and red-green double cones (white profiles); UV cones are absent from the ventral retina. The red box delimits a region used for cone cell counts. E) Retinal flat-mount from wild-type, rod reporter transgenic fish with rods (green) and cones (white profiles). F) Retinal flat-mount from *tbx2b* mutant adult with rod reporter. Scale bars: 20 µm (A,B,C,E,F) and 20 µm (D).

To visualize the planar distribution of cone photoreceptors at the surface of the retinal epithelium, we prepared flat-mount preparations of isolated adult retinas and again immunostained with anti-ZO-1 to outline the cell profiles at the OLM [10]. Figures 6A and 6C illustrate the canonical cone mosaic pattern in a wild-type, double transgenic adult zebrafish (UV and blue cone reporters, with EGFP and mCherry, respectively). Note that rows of blue and UV single cones alternate with rows of double (red/green) cones; these rows of single and double cones radiate outward from the optic disc and intersect the retinal margin orthogonally [10]. At the retinal perimeter, linear columns of new cones are generated with a precise sequence of cone identities, such that each UV cone is flanked by two green cones and each blue cone is flanked by two red cones, and the repeating units in adjacent columns are offset such that cones of the same type are never neighbors (Fig. 6C, inset). Regional variations in size of cone profiles and the relative numbers of rods in wild-type adult zebrafish alter the detailed appearance of the mosaic pattern in these preparations (compare Fig. 6A from dorsal retina and 6C from ventral retina). For example, in the dorsal hemisphere the UV cone profiles tend to be larger and the red/green double cones much smaller compared with the same cone types in the ventral hemisphere. Nevertheless, the fundamental, repeating modular unit of the cone mosaic pattern is invariant across the retina.

The crystalline lattice organization of cones is absent from the *tbx2* adult retina (Fig. 6B, D). Figure 6B shows the dorsal retina in a *tbx2b* mutant, from a region near the margin that contains a few, dysmorphic UV cones, visualized with the UV cone reporter. Note that the ZO-1 profiles that represent the non-UV cone types are not aligned in a regular, linear array. Figure 6D shows an image of ventral retina in a *tbx2b* mutant with UV and blue cone reporters. No UV cones are present in this region but blue cones are labeled. The other cone profiles outlined by ZO-1 are presumed to be red or green cones; we verified the identity of red and green cones in *tbx2b* mutants by immunostaining red/green cones with the specific monoclonal marker, zpr1 (Fig. 5B, C). The red and green cones in *tbx2* mutants are sometimes paired as double cones with flattened membrane profiles at the adjoining interface, especially in dorsal retina (Fig. 6B), but in many cases they instead form curved ‘chains’ of three or more cones with flattened adjacent interfaces (Fig. 6D).

To determine whether the correct ratio of blue to red/green double cones is generated in the *tbx2b* mutants, despite the loss of UV cones, we counted double cone profiles outlined by ZO-1 and blue cones labeled with the transgenic blue cone reporter in retinal flat-mounts of wild-type and *tbx2b* mutant adults (*e.g.*, Fig. 6D). The idealized cone mosaic pattern in adult zebrafish creates a ratio of 1 UV: 1 blue: 2 red: 2 green cones, or four red+green to one blue cone (Fig. 6C, inset). For this analysis, counts were made from samples taken from ventral retina; we did not sample dorsal retina, because in that region the red and green cone profiles are smaller and often not distinct from rod profiles in the absence of a transgene marker. In wild-type fish, these counts confirmed that the ratio of red+green to blue cones is 4.00±0.12 (mean, ±1 standard deviation, n = 7 samples from 3 retinas). In the *tbx2b* adult mutant retinas, the ratio of red+green to blue cones was 4.03±0.31, n = 7 samples from 4 retinas, p = 0.46. We therefore conclude that the relative proportions of the non-UV cone types are unaltered in the *tbx2b* mutant.

Rod photoreceptors, which are generated by proliferating progenitor cells in the differentiated retina and integrated into the epithelium as a consequence of normal growth [14], [42], [43], are largely restricted to the interfaces between cone columns (Fig. 6C, inset). This is most easily visualized in the transgenic rod reporter line, as shown in Figures 6E, F. An exception is that in older fish in some retinal regions where rods are especially abundant, they sometimes surround UV cones, inserting between the UV cone and adjacent green cones *within* a column (an example is circled in Fig. 6C). In the *tbx2b* mutant, rod photoreceptors are confined to intervening spaces between short, curvilinear chains of cones a single-cell wide (Fig. 6F); this pattern is consistent with the suggestion that these chains represent column fragments, which were generated synchronously as cohorts of new cones at the retinal margin and remain associated, as described next.

### Column fragments in *tbx2b* retina indicate directional adhesive interactions

In our previous analysis of cone lattice patterning in the zebrafish retina [10], we showed that introducing planar polarized adhesive interactions between neighboring cones into the mathematical model could reproduce the columnar cone packing observed in the biological samples. Polarized cell-cell adhesion between neighboring cone cells in zebrafish retina is mediated by the Crumbs complex [19], as we discuss further in the next section. Here we argue that, although global packing order is lost in *tbx2b* mutant adult retinas, the visible column fragments consisting of several cones chained together indicate that interactions between the cone cells remain highly directional, suggesting that PCP at the level of individual cones is not lost. Indeed, if cone-cone interactions were completely unpolarized, one would expect to see rounded clusters of cones much more often than they actually appear.

**Figure 7:**
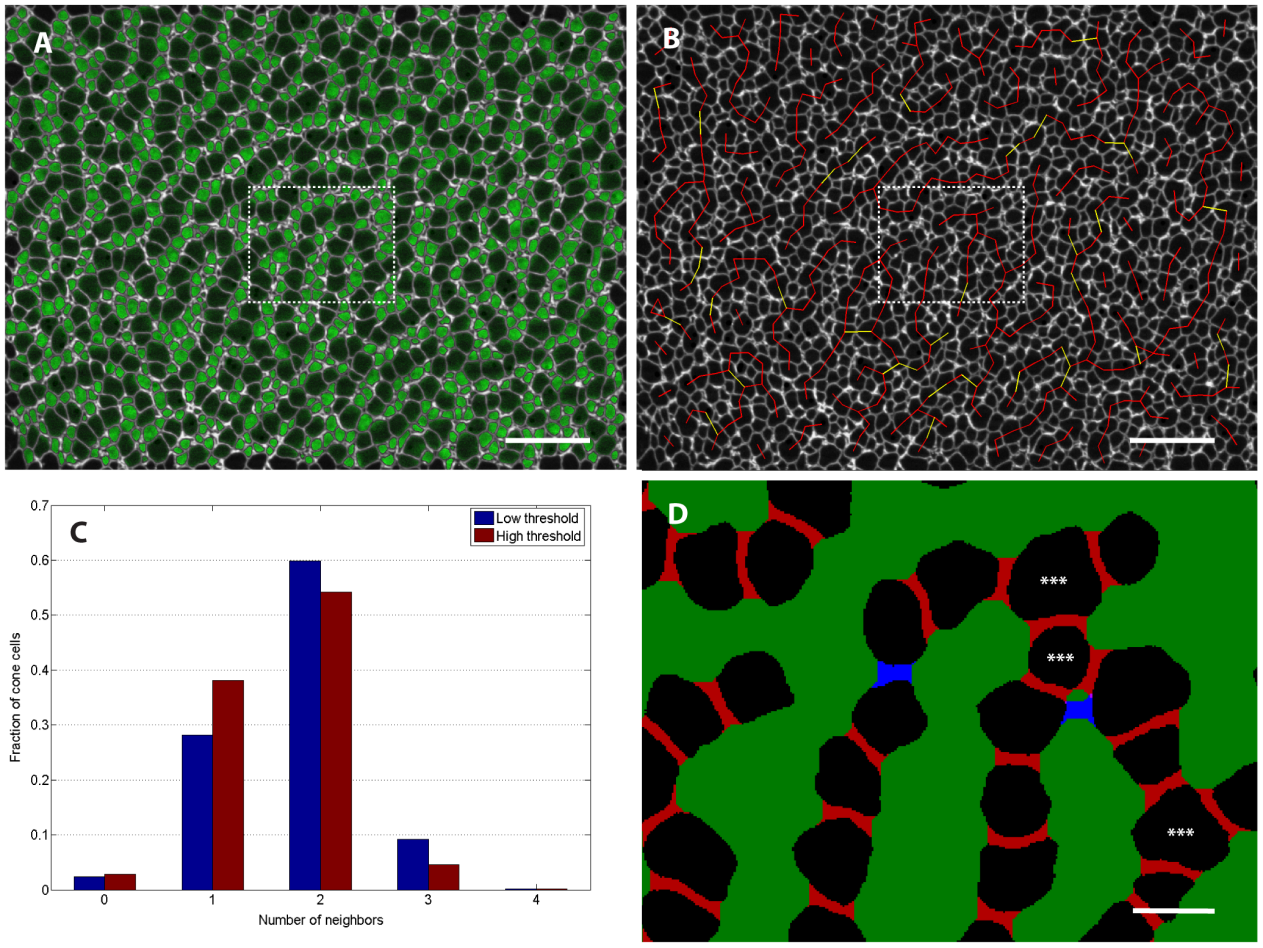
Infrequent three-fold coordination of mutant retina cone photoreceptors suggests strongly directional interaction. (See Methods section for details of image processing.) A) To identify rods, we superimposed on the ZO-1 label (white) the signal in the rod GFP reporter channel (green) from a single z-slice per cell chosen to coincide with the level of the OLM. B) Same retina as panel A. Adjacent cone photoreceptors in ventral-temporal retina of adult *tbx2b* mutant. Red lines join pairs of cones that are classified as adjacent at both low and high threshold (see Methods), while yellow lines indicate pairs that were identified as adjacent only with the less stringent threshold. (C) Histogram showing the fraction of cone photoreceptors with the specified number of identified neighbors in a sample of ventral-temporal retina. (See Table 1 for additional data.) D) Segmented image from the region outlined by the dashed box in panels A and B. Pixels between adjacent cells that were filled in by the morphological closing procedure are colored either red (high threshold) or blue (low threshold); at this magnification, 1 pixel corresponds to 0.1 µm. The two cells flanking each red region were classified as adjacent at the high threshold, but the blue region is too long (along the axis joining the centers of the two cells) and too narrow (along the orthogonal axis) to meet the adjacency criteria at the high threshold. The blue region meets the adjacency criteria at the low threshold. Stars indicate cone cells that are three-fold coordinated, *i.e.* have 3 adjacent cells. Scale bars are 20 µm for A and B, and 4 µm for D.

To quantify the extent to which cone photoreceptors in the *tbx2b* adult retinas are indeed found primarily in column fragments, we performed quantitative image analysis on several mutant retinas. This involved segmenting the composite ZO-1 image to identify the photoreceptor cells and then using the rod reporter signal to distinguish rods from cones (Fig. 7A). We next sought to formalize the intuitive observation that the cone cells appear mostly as column fragments that are one-cell thick and separated by lines of rod cells. Roughly speaking, cells should be considered adjacent along a column if they are next to each other and a share a ZO-1 stained interface of reasonable length, without intervening rods. If most cells have two or fewer neighbors by this definition (that is, they have a *coordination number* of two or less), then the epithelial cell packing is indeed dominated by a sort of columnar order suggestive of planar-polarized interactions. As described in Methods, we gave this idea of cone adjacency a more precise meaning by defining cones as adjacent if the set of pixels between them was neither too thick (along the axis joining the centers of the two cells) nor too narrow along the orthogonal axis (Fig. 7D). The definitions of “too thick” and “too narrow” are necessarily somewhat subjective. To overcome this limitation, we used two sets of parameters. The first set of parameters, corresponding to lower threshold or looser adjacency criteria, is designed to pick up most cone pairs that are likely to be adjacent, but might also include several pairs that are non-adjacent. On the other end of the spectrum, the second set of parameters corresponds to high threshold or stricter adjacency criteria, chosen to exclude most pairs that are non-adjacent with the risk of failing to include legitimately adjacent pairs. Performing our adjacency analysis as described using these two thresholds then gave us a reasonable bounds on the fraction of cones with given coordination numbers.

Figure 7B illustrates a sample result of this two-threshold adjacency determination, with red lines joining pairs of cones that were found to be adjacent at both low and high threshold and yellow lines joining pairs that were found to be adjacent only in the low threshold case. Figure 7C shows the histogram of cone photoreceptors with different numbers of neighbors from the image in panels A and B; a total of n = 454 identified cone cells were included. We found that, independent of the threshold chosen, the majority of cones are 2-fold coordinated (*i.e.*, closely adjacent to two other cones in a single-cell-wide chain) and only a small fraction of cones have higher coordination number. The asterisk in Figure 7D indicates a 3-fold coordinated cone, *i.e.* the single-cell-wide column branches. We repeated the same analysis for multiple regions in *n* = 4 different retinas; the results are tabulated in Table 1.

**Table 1:** Most cones are 2-fold coordinated in adult *tbx2b* mutant retinas.

		#	% with *n*-fold coordination (low/high threshold)				
Sample	Region	Cones	n = 0	n = 1	n = 2	n = 3	n = 4
1	D	683	31.0/43.3	50.7/47.1	17.3/9.2	0.9/0.3	0.1/0.0
2	N	493	2.6/3.0	34.3/39.1	56.8/52.7	6.3/5.1	0.0/0.0
3	T	865	7.1/13.8	37.0/46.9	45.9/35.3	9.7/3.7	0.3/0.3
4	VN	454	2.4/2.9	28.2/38.1	60.4/54.6	8.8/4.2	0.2/0.2
5	VN	677	1.5/2.1	22.7/28.4	51.0/51.0	24.1/18.0	0.6/0.6
6	VN	644	1.9/3.1	29.0/33.5	62.4/58.7	6.7/4.7	0.0/0.0
7	VN	765	5.9/7.6	51.5/56.1	37.8/33.6	4.7/2.7	0.1/0.0
8	VT	659	1.8/2.3	24.9/30.8	50.8/52.0	21.5/14.4	0.9/0.5
9	VT	840	1.5/4.2	18.5/26.1	53.1/53.5	25.6/15.6	1.3/0.7
10	VT	746	1.2/1.7	16.4/21.3	51.9/52.5	29.5/24.0	1.1/0.4
11	VT	932	1.7/2.1	22.0/31.1	51.6/48.9	22.2/16.7	2.5/1.1

Images of flat-mounted retinas from two mutant *tbx2b;rh1:EGFP* adults were processed as illustrated in Figure 7. Regions sampled included dorsal (D), nasal (N), temporal (T), ventral-nasal (VN), and ventral-temporal (T). The average % n-fold coordination with low and high parameter thresholds are given.

Table 1 illustrates the significant regional variability that we observed across the retina. For instance, we see a relatively high fraction of cones with 3-fold coordination in the ventral-temporal region. On the other hand, the dorsal retina tended to have large rod profiles and very short cone column fragments, consisting of only 1 to 3 cones each, which translated to very high fractions of 0-fold and 1-fold coordinated cones.

In summary, the relatively high fraction of 2-fold coordinated cone cells and low fraction of cones with more than two neighbors is indicative of directional adhesive interactions at the cellular level. This suggests that, although global columnar order is lost in *tbx2b* mutant retina, individual cone photoreceptors still maintain PCP in their adhesive interactions with neighboring cones.

### Planar cell polarity of Crumbs localization in cones is retained in the *tbx2b* mutant

**Figure 8:**
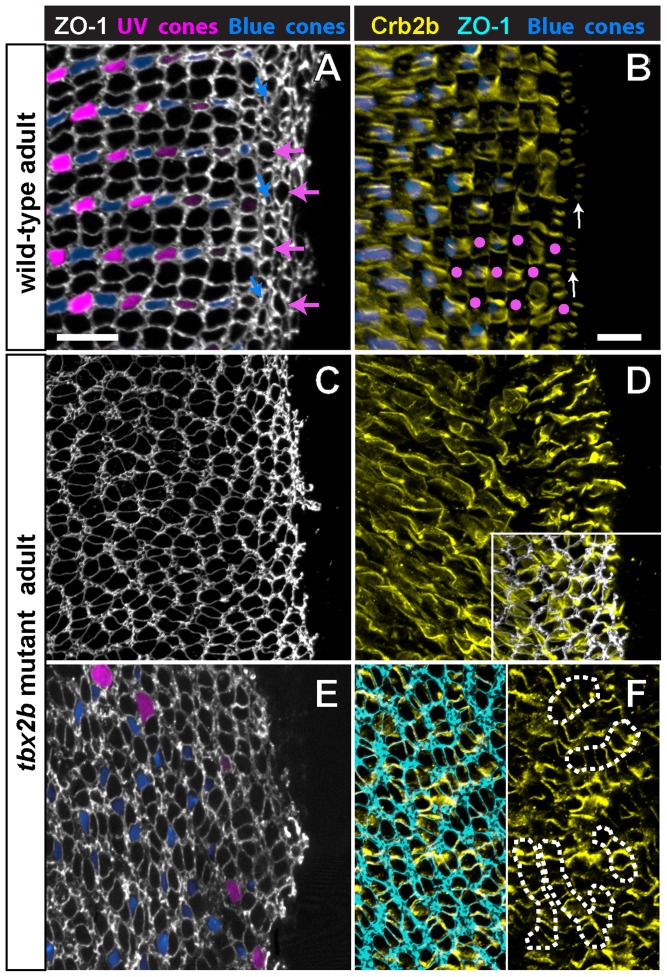
Planar polarized distribution of Crumbs2b-mediated adhesion organizes differentiating cones into linear columns, and column fragments are retained in *tbx2b* mutants. A, C, E) Confocal images of retinal flat-mounts immunostained for ZO-1 (white or cyan) and focused at the level of the OLM. B, D, F) Confocal images of retinal flat-mounts immunostained for Crb2b and focused at the level of the inner segments of cone photoreceptors. A) Retinal margin from a wild-type adult double transgenic (UV and blue cone reporters); proliferative germinal zone at the right edge. Cohorts of cones generated nearly synchronously at the germinal zone form straight columns; rows of blue cones and UV cones alternate with rows of red/green double cones (ZO-1 profiles). Expression of the blue cone reporter (blue) is initiated in differentiating cones (blue arrows) as the column emerges from the germinal zone. Cone profiles in the predicted position of UV cones (magenta arrows) emerge from the germinal zone prior to onset of the UV cone reporter. B) Retinal margin from a wild-type adult with the blue cone reporter; some UV cones, identified by their position in the mosaic lattice, are indicated by magenta dots. Crb2b immunostaining (yellow) is localized to the flattened boundaries between inner segments of five adjacent cones (green-red-blue-red-green) along a column, but not between columns; UV cones (magenta dots) are in the gaps separating the pentamers. Polarized Crb2b is in linear segments oriented orthogonal to the retinal margin in the earliest cone columns (arrows). C, D) Retinal margin from a *tbx2b* mutant double immunolabeled for ZO-1 (C) in white and Crb2b (D) in yellow; inset shows overlay of ZO-1 and Crb2b. Note that Crb2b is already polarized approximately orthogonal to the margin in the earliest cones. E) Dorsal retinal margin from a double transgenic (UV and blue cone reporters) *tbx2b* mutant adult with ZO-1 (white). F) Double immunolabeled *tbx2b* mutant. Left panel: ZO-1 (cyan) at the level of the OLM superimposed on Crb2b (yellow) at the level of the inner segments (3–6 µm apical to the OLM). Right panel: Level of Crb2b only. Dashes outline column fragments. Scale bars: 10 µm (A) and (B–F).

At the circumferential germinal zone, cohorts of newly generated cones in a linear, column a single cell wide (more precisely an annulus, when considering the entire retinal hemisphere) are added appositionally to the existing cone lattice [10]. The ZO-1 profiles of differentiating cones as they emerge from the germinal zone at the retinal margin are recognized by their larger size, rounded shape, and regular arrangement compared with the polygonal, heterogeneous profiles of the proliferating progenitor cells. The origin of the crystalline cone lattice can be discerned at or slightly peripheral to the onset of the blue cone reporter (Fig. 8A, blue arrows) and a few columns peripheral to onset of the UV cone reporter. The flattened interface that characterizes red-green double cones is also apparent within the first few columns.

The Crumbs complex – transmembrane Crumbs protein and its associated intracellular scaffolding proteins – localizes to apical membranes and is important in maintaining apical-basal polarity of photoreceptors and the integrity of adherens junctions at the OLM [3], [23]. In zebrafish, Crumbs2a (Crb2a) is expressed by rods, cones, and Müller glia, but Crumbs 2b (Crb2b) is expressed exclusively by red, green, and blue cones; the extracellular domains of the Crumbs proteins mediate selective, cell-cell adhesion between adjacent cones [19]. At the level of the cone inner segments, sometimes referred to as the subapical region [3], [10], Crb2a exhibits planar polarized distribution that is aligned with the rectangular cone lattice – higher levels of Crb2a are found at the cell-cell interface between adjacent cones *within* the columns, whereas lower levels are found at the boundaries *between* columns, where rod photoreceptors insert into the lattice [10]. This hallmark of PCP exhibited by cone photoreceptors appears at the retinal margin synchronous with the differentiation of each new cohort (column) of cones and the emergence of the cone lattice.

Here we show that similar to Crb2a, the polarized distribution of Crb2b in the apical plasma membrane at the level of the inner segments of red, green, and blue cones appears as soon as differentiating cones emerge from the germinal zone at the retinal margin (Fig. 8B and Movie S4). In the differentiated retina, Crb2b distribution in the apical membrane is localized to interfaces between adjacent blue, green, and red cones *within* columns, with gaps representing the UV cones, which do not express Crb2b (Fig. 8B). The apical cone interfaces at the boundaries *between* cone columns lack Crb2b immunoreactivity. This can be best appreciated in 3D reconstructions of the Crb2b immunolabeling (Movies S5 and S6). At the retinal margin, where the cone lattice emerges, apically polarized Crb2b creates a ladder-like pattern of staining confined to a single cone column (arrows) forming repeating units of five cones: green-red-blue-red-green [19]. An intervening UV cone that does not express Crb2b separates each pentameric unit.

The planar polarized localization of Crb2a and Crb2b proteins in the inner segment membranes of red, green, and blue cones is retained in the *tbx2* mutant retinas. At the retinal margin in the *tbx2b* mutant, as cones differentiate, a coherent column is never formed, but instead curvilinear column fragments emerge from the proliferating progenitors in the germinal zone (Fig. 8C, E). At the retinal margin, a roughly ladder-like pattern of Crb2b can be discerned (Fig. 8D), but without the strictly parallel orientation of the Crb2b interfaces seen in the wild-type at the retinal margin. Within differentiated regions of the *tbx2b* mutant retina, Crb2b is polarized to the interfaces between adjacent cones in the single-cell-wide column fragments. Figure 8F (and Movie S7) illustrates a double-immunostained retinal flat-mount preparation: ZO-1 identifies column fragments at the level of the OLM, and more apically at the level of the inner segments, Crb2 is localized to the boundaries between adjacent cones in a column fragment.

**Figure 9:**
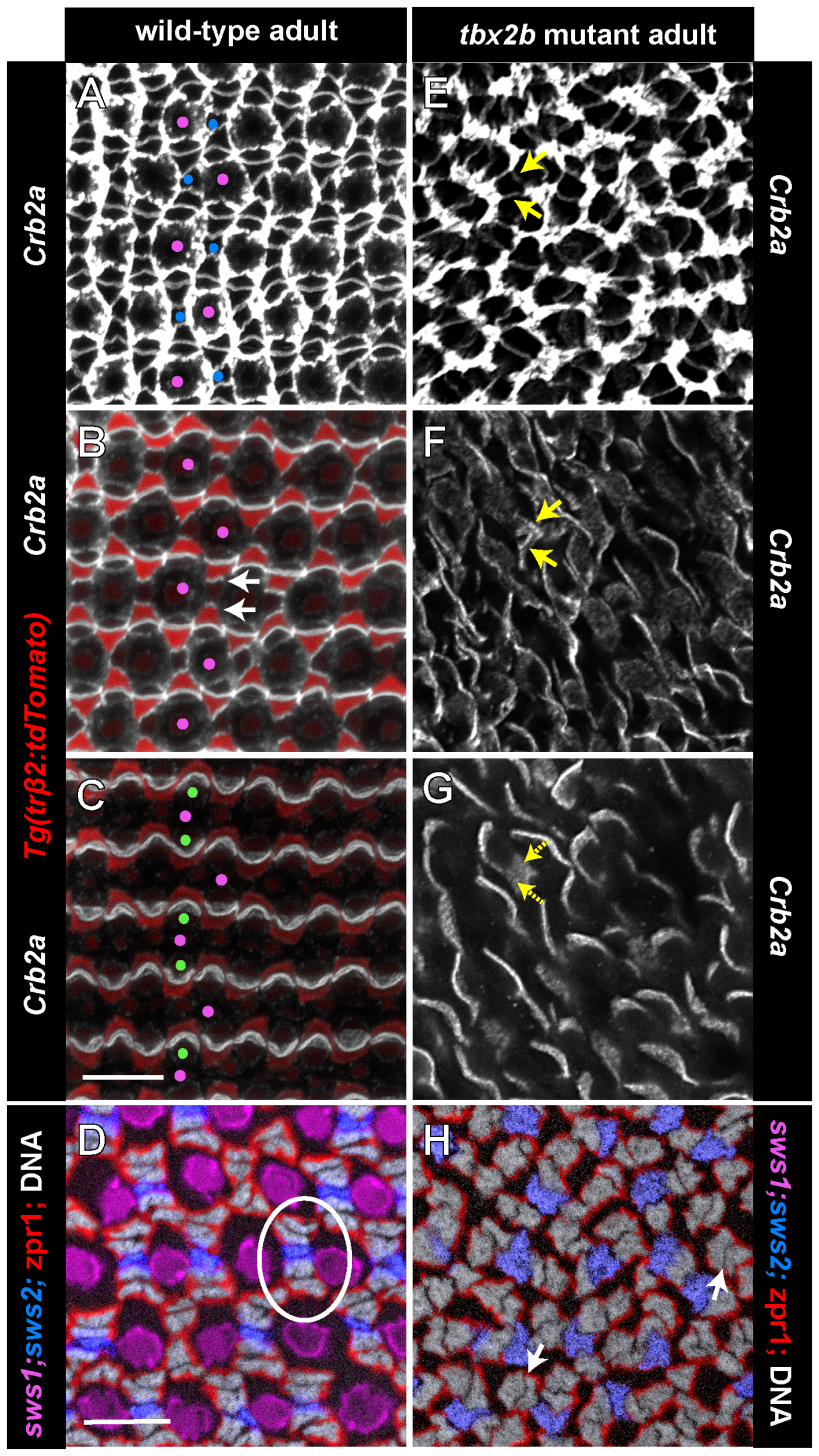
Planar polarized distribution of Crumbs2a is retained at the level of cone inner segments in *tbx2b* mutants despite loss of pentameric organization of red, green, and blue cones. Panels A–C and E–G are confocal images of retinal flat-mounts immunostained for Crb2a (white) in a wild-type, transgenic (*trβ2:tdTomato*) fish (A–C) in which red cones express the tdTomato reporter (red), and a *tbx2b* mutant (E–G), which expressed the *sws1:EGFP* reporter, although no UV cones are present in the region imaged. Each panel is a projection of 3 to 12 optical sections selected from a complete, vertical z-stack of the Crb2a immunolabeling: panels A, E are at the level of the OLM; panels B, F are 2–4 µm apical to the OLM, at the level of the red, green, and blue cone inner segments; panels C, G are 6–7 µm apical to the OLM, at the level of the red and green inner segments. A) Selected UV and blue cones are identified with magenta and blue dots, respectively, based on their relative sizes and positions in the mosaic array at the OLM. B) At the level of inner segments, Crb2a is preferentially localized to interfaces between red/green double cones and between a blue cone and the two flanking red cones (arrows). The inner segments of UV cones (magenta dots) are short and do not extend this far apically. C) Only the longest cones (red/green double cones) extend to this level, and Crb2a remains polarized. E) Retinal flat-mount from a *tbx2b* mutant at the OLM. This mutant did not carry the *sws2:mCherry* transgene, so the blue cones cannot be distinguished from the red and green cones. F, G) Short, curved segments of Crb2a, which resemble red/green double cone interfaces in the wild-type extend to the most apical level (G), but other Crb2a segments (yellow arrows) are at the intermediate level (F) but not higher (G). These may represent blue cones. Panels D and H show tangential cryosections through the level of nuclei of red, green, and blue cones; nuclei are stained with Hoechst (grey); red and green cones are immunolabeled with the membrane-associated, specific antibody, zpr1 (red). Both the wild-type (D) and *tbx2b* mutant (H) fish carried the UV and blue cone transgenes, *sws1:EGFP* (magenta) and *sws2:mCherry* (blue), although this retinal region lacked UV cones in the mutant. A single pentameric unit is encircled by an oval (D) and white arrows point to the interface between red and green double cone pairs. Scale bars: 10 µm (A, B, C, E, F, G) and (D, H).

All retinal cells that extend through the apical surface (*i.e.*, rods, cones, and Müller glia) express Crb2a, which outlines cellular profiles at the level of the OLM (Fig. 9A). In the apical plasma membrane, Crb2a co-localizes with Crb2b in a planar polarized pattern at the interfaces between adjacent red/green double cones and between red cones and adjacent blue cone inner segments *within* columns (Fig. 9B, C). In the *trβ2:tdTomato* transgenic line, the reporter identifies red cones, whose inner segments curve around their green cone partners (Fig. 9B,C). The inner segment processes of UV cones are much shorter than the other cone subtypes (Fig. 5A), and they do not participate in these apical adhesive interactions [19]. In the *tbx2b* mutant, Crb2a also outlines cell profiles at the OLM (Fig. 9E), and is polarized at cone-cone interfaces at the level of the inner segments (Fig. 9F, G) similar to Crb2b (Fig. 8D). The fragmentation of cone columns in the mutant disrupts the long-range orderliness of these boundaries. In the wild-type, two pairs of red/green double cones, identified with zpr1 staining, flank each blue cone within a column to form pentameric units, one of which is encircled with a white oval in Fig. 9D. In the *tbx2b* mutant, pentameric units are not found (Fig. 9H); although some profiles resemble red/green double cone pairs (white arrows), other zpr1-labeled cones are unpaired, and the positions of blue cones with respect to red and green cones are not consistent.

### The coherent lattice pattern of the remaining cones remains intact when UV cones are selectively ablated in the adult retina

**Figure 10:**
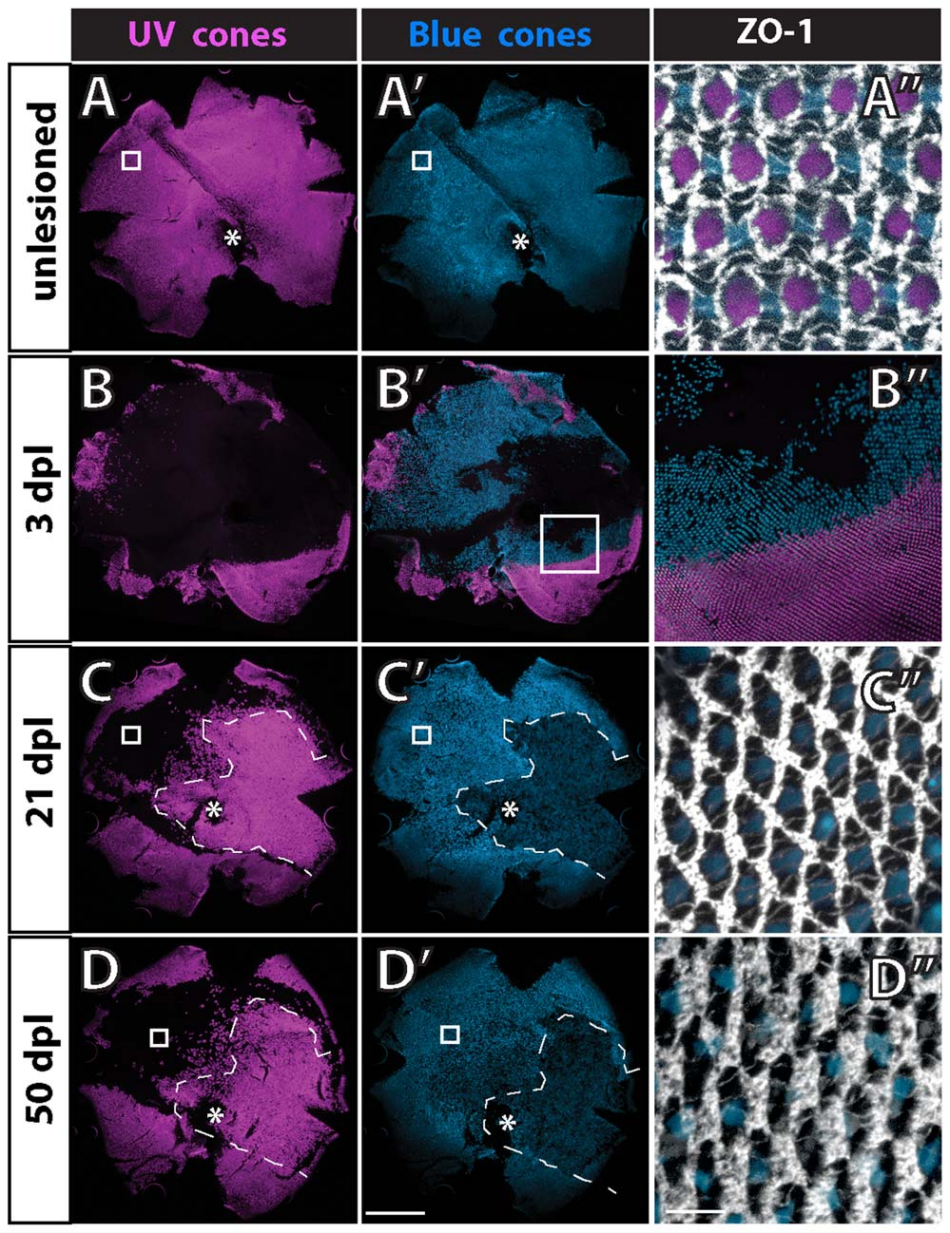
The mosaic pattern is not disrupted after selective ablation of UV cones in adult wild-type fish. Retinal flat-mounts from a double transgenic adult fish (UV and blue cone reporters) immunostained for ZO-1. Ventral is down; optic disc (*); boxed area in panels to the right. A) Control retina, UV cones; A′) blue cones; A″) ZO-1, UV and blue. B) Transgenic fish exposed to intense light, 3 days post-lesion (dpl), UV cones; B′) blue and UV cones; B″) boundary of ventral region in which no cones are ablated (lower right) and only UV cones but not blue cones are ablated (upper right). C) At 21 dpl, all cone types, including UV cones, were ablated and have regenerated within the central and temporal retina in the region enclosed by dashes; C′) blue cones have regenerated within the region enclosed by dashes; C″) in the boxed region where only UV cones were ablated they do not regenerate. D) At 50 dpl, UV cones (along with other cone types) have regenerated within the region enclosed by dashes; D′) blue cones have regenerated within the region enclosed by dashes; D″) rods continue to accumulate in the spaces previously occupied by UV cones in the region where they were selectively ablated and fail to regenerate. Scale bars: 500 µm (A, A′ through D, D′); 10 µm (A″ through D″).

In contrast to the disrupted cone pattern in the *tbx2b* mutant, in which UV cones largely fail to differentiate and when they do they are dysmorphic, selective ablation of UV cones from the adult retina does not disrupt the pattern of the cone mosaic, which retains its rectilinear organization. Photoreceptors in adult zebrafish can be destroyed by several minutes of exposure to intense light [31], and UV cones are especially sensitive. Where all cone types are destroyed, in a region of retina along the horizontal meridian (Fig. 10A, B), cones regenerate within a week [31], although, as predicted, the regular lattice pattern is not reestablished [10], [44]. In some areas of dorsal retina, UV cones are destroyed by the light, but red, green, and blue cones are spared (Fig. 10B). Where the UV cones are selectively ablated, they are not regenerated, even after several weeks, although the remaining cones retain their regular lattice pattern (Fig. 10C, D). The abrupt elimination of UV cones from the adult retina somewhat deforms the shape of the green-red-blue-red-green pentameric units, which are constricted at each end (Fig. 10C), but with increasing time after the lesion, rod photoreceptors accumulate in the vacant spaces where UV cones previously resided, and within weeks the rectilinear cone pattern is fully restored (Fig. 10D).

### A mathematical model that incorporates elimination of UV cones reproduces the disrupted cone pattern seen in *tbx2b* mutants

As mentioned above, we noticed that at the retinal margin, expression of the UV opsin reporter lags behind that of the blue opsin reporter (Fig. 8A, blue arrows) by at least one column cohort; however, large, round cone profiles in the predicted position of UV cones are easily discerned in the earliest cone column and sometimes more peripherally (Fig. 8A, magenta arrows). Expression of opsin is a relatively late event in photoreceptor differentiation [45], [46], [47], and cone progenitors are specified and initiate differentiation prior to expression of the transgene opsin reporters. In the developing, embryonic goldfish retina [45] as well as at the growing retinal margin in adult goldfish [48] the onset of UV opsin is delayed compared with the other cone opsins. These observations together suggest that UV cones are committed and begin to differentiate before the UV opsin reporter is expressed.

As described above, *tbx2b* mutants lack the crystalline order of the wild-type cone mosaic pattern, although fragments of cone columns persist with some cones having three or more neighbors. The unusual distribution of residual UV cones in the *tbx2b* mutants (Fig. 3), the absence of obvious apoptosis, the emergence of cone profiles at the retinal margin in the predicted location of UV cones in advance of a fully-formed cone column (Fig. 8A), and the abnormal morphology and subsequent loss of residual UV cones in the dorsal retina (Fig. 5B,C) lead us to speculate that in the *tbx2b* mutant UV cones might originally be specified at the retinal margin, but then removed from the epithelium, perhaps because of defects in their program of differentiation. The mechanism of elimination could be apoptosis, although neither TUNEL nor anti-caspase immunoreactivity provided direct evidence for cell death. Another mechanism known to eliminate cells from epithelia, either with or without apoptosis, is a process of active cell extrusion [39], [40]. We therefore investigated whether the mathematical model we developed previously, which reproduces the salient features of the establishment of the cone mosaic pattern at the retinal margin as seen in the wild-type fish [10], could also predict the features of the pattern disruption seen in the *tbx2b* mutant if we incorporated elimination of committed, but not fully differentiated, UV cones at the retinal margin. We also examined the implications for patterning if PCP proteins are present in the dysmorpic UV cones.

The model [10] describes the epithelium as a two-dimensional, planar array of cells that meet at junctional complexes (*edges*); the length of an edge is determined by a balance of cell-adhesion and cortical acto-myosin contractility that can be accounted for by introducing an effective mechanical tension along each edge. The model incorporates a bidirectional interaction between the PCP proteins and anisotropic stresses acting on the mechanical packing that deform the cell shapes. The PCP proteins that mediate cell-cell adhesion and regulate acto-myosin contractility at the adherens band (*e.g.*, Crumbs complex) accumulate on the shorter cell edges, and the tension on a given edge depends on the localization of the PCP proteins on it – high tension edges have a low concentration of PCP proteins and vice versa, thereby further influencing the cell shape. Since the zebrafish retina grows by adding a new column of cones at the retinal margin, as described above, this progressive growth is mirrored in the model by changing a column of precursor cells, initially devoid of PCP proteins and in contact with an ordered column of cones, into a new column of polarized cone cells. The cells belonging to the new column are initially given a random distribution of PCP proteins, which eventually redistribute on the edges to establish a polarized direction. Hence, this model showed that the establishment of cone mosaic in wild type retina requires a coupling between the anisotropic stresses and PCP proteins, as well as a propagating front of newly formed cone cells.

To model the disordered cone pattern in the *tbx2b* mutants, we consider the effect of selective elimination of UV cones in the newly established column of cones at the retinal margin. In the model, uniformly spaced cells in the new column were marked as UV cones, such that approximately five red, green, or blue cones lie between two successive UV cones, as seen in the wild-type retina (Fig. 6C, inset). Studies on overcrowded epithelia in *Drosophila* dorsal notum have shown that local changes in mechanics play a major role in driving cell extrusion [49]. In this system, extruding cells progressively lose edges while simultaneously losing apical area. Other studies, including in zebrafish epidermis [50], have shown that the apical extrusion of a cell is preceded by contraction of the actin-myosin ring in the neighboring cells around it [39], [40]. The effects of epithelial cell apoptosis on the packing of the remaining cells have not been systematically characterized, but it seems likely that a similar, relatively rapid contraction at the level of the adherens band is involved [50].

**Figure 11:**
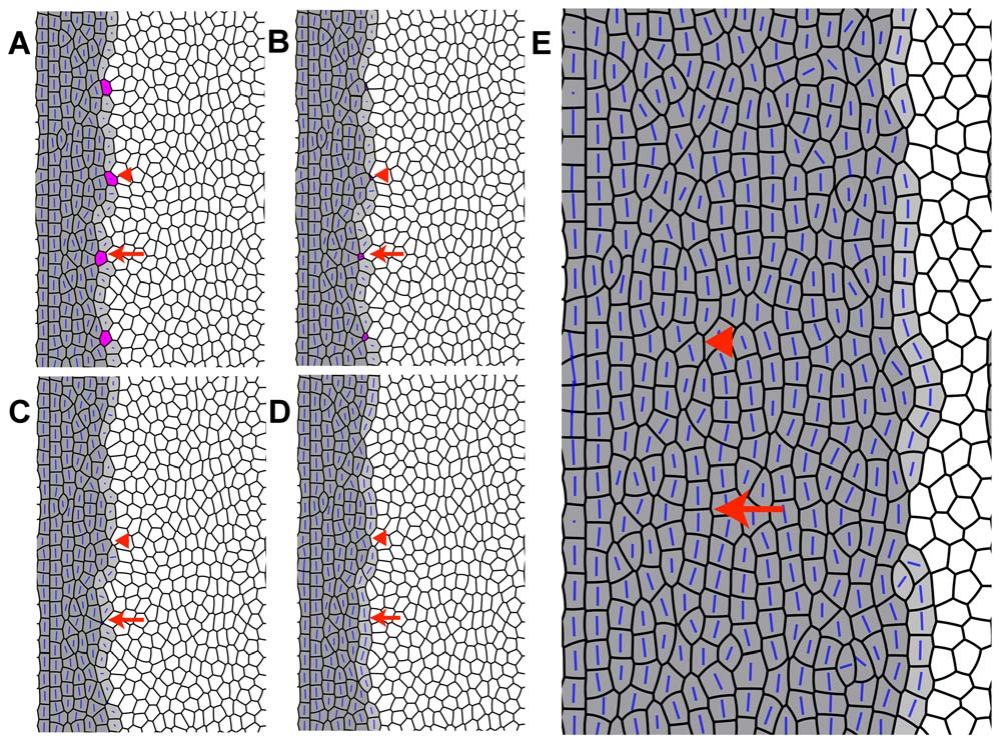
Disordered cone packing obtained from simulations. A, B, C, D) Sequence of events after loss of presumptive UV cones (represented as magenta cells) from the column of new cones (light grey cells) that are generated by proliferating precursors (white cells) to the right. The established columns of differentiated cones are at the left (dark grey cells). Blue bars represent the direction of polarization imparted by planar cell polarity proteins; the length of the bar indicates the strength of planar polarization within the cell. The elimination of UV cones is modeled by loss of cell edges via successive T1 transitions with a simultaneous decrease in cell area (see Methods for details). The arrowheads point to an example in which loss of the UV cones breaks the column such that the precursor cells are interspersed between the differentiating (grey) cone cells. The arrows point to an example in which the column is left intact, forming long single-cell wide column fragments. E) After successive columns of new cone cells are added by the propagating front (moving from left to right), accompanied by loss of UV cones, disordered cone packing results, with small column fragments that are not aligned.

Taking a cue from these studies of epithelial cell extrusion, in our model we increase by threefold the tension along the edges of the presumptive UV cones in the propagating front. These UV cones are also subjected to an internal negative pressure, which leads to their shrinkage in the plane of the cell packing by subsequent loss of edges via topological transitions known as T1 transitions [35] and eventually to their elimination. Under slow relaxation, the cell shape of the shrinking UV cone is deformed by the polarization of the PCP proteins. The edges with higher tensions are lost first and hence the adjacent cells within the column expand and occupy the vacated space, leaving the columns intact. However, when the relaxation time is short, the UV cells shrink fast regardless of the polarization direction of the proteins, thereby rendering arbitrary the order in which edges are lost (Fig. 11 A–C). This can either leave the column intact resulting in the formation of longer, single-cell wide cone columns (marked with an arrow in Fig. 11) or lead to a break in the column with a precursor cell infringing on the cone column (marked with an arrowhead in Fig. 11), with the latter outcome being more prevalent (∼68%, *n* = 300 eliminated UV cones). When subsequently the next column of cones is established, a merging of columns occurs, as shown in Fig. 11E, wherein the neighboring blue cones in the adjacent columns expand to occupy the space vacated by the UV cones. When these defects accumulate with progressive growth of the mosaic, the final pattern resembles the disordered cone patterning seen at the retinal margin in *tbx2b* mutants (Fig. 8B, C) with many column fragments consisting of five or more cells (see Movie S8, for the complete simulation sequence).

Our simulation suggests the following: 1) When the elimination of a UV cone occurs on a very short timescale, column fragments are formed, leading to a disrupted pattern of cone cells. 2) PCP proteins can play a role in maintaining order when the elimination of UV cones occurs on a timescale comparable with the relaxation time of the PCP proteins. Note that this model does not include rods. Whereas in *tbx2b* mutants column fragments ultimately are interspersed with rods, which insert themselves in the spaces between column fragments, rods are not such a prominent feature near the margin (Fig. 8C and [10]), suggesting they are not essential to the mechanism of cone mosaic disruption.

## Discussion

The self-organization of cone photoreceptors into geometrically precise patterns of specific cone types within the epithelial plane of the retina is a hallmark of retinal development in teleost fish, yet the details of how this is accomplished remain largely unknown. In this study we used a zebrafish genetic model in which UV cone photoreceptors are largely missing as a result of mutations in the *tbx2* locus [25] to examine the consequences of their loss on organization of the cone mosaic pattern. Tbx proteins are transcriptional regulators and members of a large, ancient, diverse family of T-box genes that are expressed in highly specific spatiotemporal patterns and have important roles in embryogenesis, including cell fate decisions that establish the early body plan, as well as later involvement in tissue polarity and organogenesis of the heart, limb, and other organs [51]. Tbx proteins interact with and regulate multiple signaling pathways (including BMP, FGF, Hedgehog, Wnt, and retinoic acid), and misregulation of *tbx* genes is associated with defects in cell cycle control, human developmental dysmorphic syndromes, and neoplastic processes [51]. Tbx2 has been implicated in several developmental processes involving coordinating cell fate, patterning and morphogenesis in several tissues, including the brain [30], [52] and the eye [25], [53], [54]. The zebrafish Tbx2 subfamily is expressed in the early optic primordia, otic placodes, other sensory placodes and cranial nerves, sensory and motoneurons in the spinal cord, primorida of the kidney, heart, liver [55].

Tbx2b is known to be involved in establishing the dorsal/ventral axis of the retina in both mice [53] and zebrafish [54] although the mechanistic details are uncertain. In zebrafish, *tbx2b* expression first appears throughout the retinal neuroepithelium at 15 hours post-fertilization (hpf), but by 24 hpf expression is mostly restricted to the presumptive dorsal retina [54] and is largely absent from the ventral region adjacent to the choroid fissure [54]. The region that lacks *tbx2b* corresponds to the precociously differentiating, ventral-nasal patch of retina that has an unusually high density of rapidly developing rod photoreceptors [36], [37]. Morpholino antisense knockdown of *tbx2b* results in delayed photoreceptor development, and markers specific to both rods and double cones were absent in the dorsal retina at 72 hpf, potentially signifying that photoreceptor progenitors failed to exit the cell cycle [54]. In the developing mouse optic vesicle, Tbx2 is downstream of the BMP4 signaling pathway and functions to block retinoic acid production to establish dorsal identity and regulate cell proliferation [53]. Interestingly, retinoic acid can enhance differentiation of mammalian rods in cell culture by shortening the amount of time between terminal mitosis and rhodopsin expression [56], and exposure of zebrafish embryos to exogenous retinoic acid increases the number of rods and red cones and reduces the number of UV and blue cones [57]. These results suggest that the increased number of rods we and others [18] observed in *tbx2b* mutant embryos might be caused by derepression of retinoic acid synthesis in the embryonic retina. Clearly, given the number and diversity of signaling pathways interacting with Tbx2b, the variety of phenotypes resulting from a reduction in Tbx2 function will have complex mechanistic explanations that are context dependent.

Our analysis shows that loss of Tbx2b function disrupts the differentiation of UV cones and profoundly disturbs the long-range patterning of the cone mosaic in the adult zebrafish retina – remaining cones are not organized in a crystalline lattice but instead are arranged in curved column fragments separated by rods. The defects we observed in cone patterning in the *tbx2b^c144^* mutant were correlated with a significant loss of UV cones. Tbx2b is expressed in retinal progenitors in the germinal zone [58], and although the defect in specification and/or differentiation of UV cones we documented in the *tbx2b* mutant is correlated with disruption in the organization of the cone mosaic pattern, we cannot exclude the possibility that Tbx2b has additional functions that influence the positioning of cones as they differentiate, independent of the loss of UV cones. Since the other cone types – blue cones and red/green double cones – were present in the mutant retina in the correct ratio, and they exhibited normal polarized distribution of Crb2b and Crb2a, Tbx2b must not have a generalized role in cone photoreceptor specification or differentiation.

One of the predominant features of the wild-type cone mosaic is the packing of cone photoreceptors at the level of the OLM into straight, circumferential columns parallel to the retinal margin. Within these columns, cone cells interact with each other apically via planar polarized adhesion; in particular, proteins associated with the Crumbs complex are seen to be localized to cell-cell interfaces at the level of the inner segments *within*, but not *between*, columns. We found that, although the global, rectilinear order of the cone mosaic pattern was lost in *tbx2b* mutants, the tendency to organize cone cells locally into single-cell wide, column-like structures, survived. Indeed, our quantitative analysis of the cone mosaic in mutant fish revealed that the large majority of cones made strong, direct contact with at most two other cones, consistent with a scenario in which they are found primarily in one-cell thick column fragments. Moreover, the cones in these fragments still exhibited planar polarized distributions of Crumbs proteins oriented correctly with respect to their particular column (Figs. 8,9). Thus, the blue, red, and green cones that remain in *tbx2b* mutants retain their individual capacity for directional interactions, but they have lost the ability to coordinate their polarization across the retina.

We have no evidence that would implicate rod photoreceptors as causal agents in the defective cone mosaic pattern in *tbx2b* mutants. A previous study examined the influence of Tbx2b on development of zebrafish photoreceptors with a weaker *tbx2b* allele (*tbx2b^p25bbl^*), in which expression of *tbx2b* is reduced; they reported increased rods and reduced UV cones with correspondingly high levels of rhodopsin and rod arrestin mRNA and extremely low levels of the UV opsin in mutant embryos compared with wild-type embryos [25]. In contrast, histological analysis of the photoreceptor layer in adult *tbx2b^p25bbl^* mutants, revealed no increase in rods, normal morphology of remaining UV cones, and a normal cone mosaic lattice. In the stronger *tbx2b^c144^* allele [30] that we examined here, the phenotype in the embryonic retina was similar, but in the adult retina the organization of the cone mosaic was severely disrupted. A tendency toward increased number of rods persisted (where we measured it, in ventral retina), although the difference was not as robust – an ∼30% increase in rods in the adult compared with a 2-fold increase in the embryo – and there was no significant increase in rod opsin message in the retina as a whole. Similarly, in the weaker *tbx2b^p25bbl^* allele, the excess rod phenotype largely disappeared in the adult retina [25].

Disruption of the cone lattice was specifically associated with absence or early loss of UV cones during the initial stages of cell fate commitment, differentiation, acquisition of PCP, and positioning of cone photoreceptors at the growing retinal margin, whereas light-induced, selective ablation of UV cones from the intact retina after the cone mosaic was established did not perturb the strong adhesive interactions holding the remaining cones in their places. Instead, rod photoreceptors, which persistently accumulate in the adult teleost retina as the eye grows [42], [43], filled in the gaps in the mosaic pattern where the UV cones were lost. Natural loss of UV cones without perturbing the cone mosaic pattern also occurs in some migratory salmonid fishes, in which thyroxine-mediated loss of UV cones is associated with metamorphosis in preparation for migration to deeper, marine waters [59], [60].

The inconsistency among *tbx2b* mutants in the number and distribution of UV cones suggests that other factors might regulate their production and/or survival. The persistence of UV cones in the center of the retina, and at the periphery, but only in dorsal retina, is perplexing. Since *tbx2b* mRNA is expressed at higher levels in retinal progenitors in the dorsal compared with the ventral optic primordium in the embryo, and this pattern of dorsal expression persists in the retinal progenitors in the circumferential germinal zone of the larval retina [25], [54], if the mutant protein is partially functional, perhaps UV cones in dorsal retina have sufficient Tbx2b to allow some to differentiate, albeit defectively. Alternatively, other Tbx genes that are also expressed in a gradient in the retina with higher levels dorsally – *tbx2a* [61] or *tbx5a* and *tbx5b* [58] – might partially compensate for the loss of *tbx2b* function in the mutant.

Because the zebrafish retina grows by addition of new cones at the retinal margin, in a single flat-mount preparation we can examine successive stages in formation of the cone lattice pattern as it emerges from the progenitor cells in the circumferential germinal zone. As each cohort of presumptive cone photoreceptors exits the cell cycle and differentiates, new columns parallel to the retinal margin are added to the cone mosaic lattice. UV and blue cone opsins are not expressed until relatively late during the sequential process of cone differentiation [45], [47], so the EGPF and mCherry opsin reporters we used to identify UV and blue cones, respectively, do not label differentiating cones at the earliest developmental stages. Yet it is clear that when columns of presumptive cones (identified by their ZO-1 profiles) emerge from the germinal zone in wild-type adult retinas, they are already precisely aligned with the adjacent column that immediately preceded them even before they express the opsin reporters (Fig. 8A). In the *tbx2b* mutant, fragments of cone columns extend to the germinal zone, and continuous columns aligned parallel to the retinal margin are never observed (Fig. 8C), even in those regions of the retina where UV cones have not been completely eliminated.

In our previous studies of cone mosaic formation in wild-type fish [10], we had developed a mathematical model of mosaic formation emphasizing the establishment of cone columns near the retinal margin, mediated by PCP and external mechanical stresses. We had observed that this model was particularly sensitive to perturbations when a new column of cones emerged from the proliferative germinal zone. Whereas defects introduced at this moment tended to become “quenched in” and were never corrected, once the regular lattice had been established, it was much harder to destroy it. We thus reasoned that the *tbx2b* phenotype in adult fish might be closely tied to the fact that UV cones appear to be lost very near the margin, after they are committed to a cone fate but before the UV cone reporter is expressed. The relatively few UV cones that appear transiently and only at the dorsal retinal margin are dysmorphic and they do not persist (with the possible exception of UV cones generated in the embryonic retina). We are intrigued by the report that epithelial cell extrusion is a consequence of reduced levels of Tbx2b in neural plate cells in zebrafish embryos, where Tbx2b-deficient ectodermal cells have defective cell movements, with loss of cell-cell adhesion, and subsequent exclusion from the epithelium [52]. To date we have no direct evidence to support cell extrusion as a mechanism for UV cone elimination, nor do we have evidence for selective apoptosis of UV cones in the *tbx2b* mutant retinas. Nevertheless, to study whether elimination of UV cones soon after they are specified would be sufficient to reproduce the mosaic disruption seen in *tbx2b* fish, in the absence of any direct effect on the remaining cone types, we modified our previous computational model to include removal of UV cones at the margin immediately after a new column of cone photoreceptors begins to differentiate. We found (Fig. 11; Movie S8) that, as long as either removal of UV cones occurred relatively fast, or PCP was lost as soon as UV cone shrinkage began, this model reliably produced mosaic defects very similar to those observed near the margin in *tbx2b* fish (Fig. 8C), where misaligned column fragments are seen to emerge from the germinal zone. Thus, even though they do not participate in the same tight, planar polarized contacts at the level of the inner segment as the other cones do, UV cones are an essential part of the regular cone lattice, since their loss is sufficient to destroy the crystalline order during its formation.

## Supporting Information

Movie S1 **3D animation of the retinal margin in a wild-type adult transgenic zebrafish,** ***sws1*** **:EGFP reporter for UV cones.**
(MP4)

Movie S2 **3D animation of the dorsal retinal margin in a** ***tbx2b*** **mutant adult transgenic zebrafish,** ***sws1*** **:EGFP reporter, showing dysmorphic UV cones.**
(MP4)

Movie S3 **3D animation of the dorsal retinal margin in a** ***tbx2b*** **mutant adult transgenic zebrafish,** ***sws1*** **:EGFP reporter, showing dysmorphic UV cones.**
(MP4)

Movies S4 **3D animation of ZO-1 immunostaining at the retinal margin of a wild-type adult zebrafish (single channel displayed from** **Figure 8A** **).**
(MP4)

Movie S5 **3D animation of Crb2b immunostaining (white) in a retinal flat-mount from a wild-type adult transgenic zebrafish, blue cone opsin reporter.** Viewed from the apical surface.
(MP4)

Movie S6 **3D animation of Crb2b immunostaining (white) in a retinal flat-mount from a wild-type adult double transgenic zebrafish, blue and UV cone opsin reporters.** Viewed from the apical surface.
(MP4)

Movie S7 **3D animation of Crb2b immunostaining (white) and ZO-1 immunostaining (cyan) in a retinal flat-mount from a** ***tbx2b*** **mutant adult zebrafish.** Viewed from the apical surface.
(MP4)

Movie S8 **Simulation showing the progressive growth of the cone pattern in the presence of extrusion of UV cones (shown here in magenta) in the propagating front.** A disordered cone pattern with small column fragments is obtained.
(MP4)
